# Socio-ecological impact of monogenetic volcanism in the La Garrotxa Volcanic Field (NE Iberia)

**DOI:** 10.1038/s41598-023-35072-0

**Published:** 2023-05-20

**Authors:** Jordi Revelles, Joan Martí Molist, Francesc Burjachs, Walter Finsinger, Eneko Iriarte, Francesc Mesquita-Joanes, Sergi Pla-Rabés, Llorenç Planagumà, Maria A. Rodrigo, Gabriel Alcalde, Maria Saña

**Affiliations:** 1 Institut Català de Paleoecologia Humana i Evolució Social (IPHES-CERCA), Zona Educacional 4, Campus Sescelades URV (Edifici W3), 43007 Tarragona, Spain; 2 Universitat Rovira i Virgili (URV), Àrea de Prehistòria, Avinguda de Catalunya 35, 43002 Tarragona, Spain; 3 Department of Geosciences, Institute of Environmental Assessment and Water Research (IDAEA-CSIC), Jordi Girona 18-26, 08034 Barcelona, Spain; 4 ICREA, Pg. Lluís Companys 23, 08010 Barcelona, Spain; 5 ISEM, University of Montpellier, CNRS, IRD, EPHE, Montpellier, France; 6 Laboratorio de Evolución Humana/IsoTOPIK, Departamento de Historia, Geografía y Comunicación, Universidad de Burgos, Plaza Misael Bañuelos s/n, Edificio de I+d+i, 09001 Burgos, Spain; 7 “Cavanilles” Institute of Biodiversity and Evolutionary Biology, University of Valencia, Catedrático José Beltrán Martínez, 2, 46980 Paterna, Spain; 8 Unitat d’Ecologia, Departament de Biologia Animal, de Biologia vegetal i Ecologia, Universitat Autònoma de Barcelona, 08193 Bellaterra, Catalonia Spain; 9 CREAF, Center for Ecological and Forestry Applications, 08193 Cerdanyola del Vallès, Catalonia Spain; 10 Tosca, Environment Services of Education, Casal dels Volcans, Av. Santa Coloma, 17800 Olot, Spain; 11 Departament Història i Històriadel’Art, Universitat de Girona, 17071 Girona, Spain; 12 Departament de Prehistòria Edifici B, Facultat de Filosofia i Lletres, Universitat Autònoma de Barcelona, 08193 Barcelona, Spain

**Keywords:** Environmental social sciences, Climate-change impacts, Environmental impact, Natural hazards

## Abstract

Volcanism can cause major impacts, including climate change and mass extinctions. However, the impact of monogenetic volcanism is often considered as limited in volcanological research. This work provides for the first time an interdisciplinary approach to the socio-ecological impact of monogenetic volcanism in a key region, the La Garrotxa Volcanic Field (GVF, Girona, NE Iberia), where intense monogenetic volcanic activity occurred in the past. The analyses of a sedimentary sequence from the GVF enabled identifying previously unknown volcanic eruptions in the time interval 14–8.4 ka cal BP, constrain their volcanic stratigraphy and age, and unfold the effects of environmental change on geomorphology, vegetation, aquatic organisms and humans. Moreover, we reconstruct the major palaeoenvironmental changes caused by the eruptions in terms of fire episodes and subsequent disturbance on vegetation, hydrology and limnological conditions. When put in context with the archaeological record, it appears that the last hunter–gatherer communities were resilient at an extra-local scale, facing episodes of vulnerability due to volcanic activity, suggesting that their flexible nomadic patterns and foraging economies were an efficient source of risk management against the volcanic eruptions and their ecological impacts.

## Introduction

There is ample evidence to support the view that volcanic eruptions have caused in the past major impacts on environment and societies, both directly (e.g. lava flows, tephra deposition and earthquakes^1–3^), as well as indirectly (e.g. contributing to climate change^4–6^, mass extinctions^7,8^ and disturbance on human communities^9–11^). However, volcanological and related palaeoecological research has been mainly focused on explosive eruptions from polygenetic volcanoes (with repeated eruptions), such as Laacher See (Germany^1^), Santorini (Greece^12^), Somma-Vesuvius and Campanian Ignimbrite (Italy^13,14^), Mount Pinatubo (Philipines^15^) or Toba and Krakatoa (Indonesia^16^), but the impact of monogenetic (single small basaltic eruptions) volcanism has been generally neglected or considered as limited.

The La Garrotxa monogenetic Volcanic Field (GVF) is the youngest (Middle Pleistocene (*ca*. 350 ka BP) to early Holocene) volcanic area of the Iberian Peninsula. Recent studies in this area have addressed the characterization of the volcanic activity and petrology of volcanic products^17–21^ and the geological and structural controls of the magmatism and related volcanism^22–24^. Still, the impact of these eruptions on the environment and past human societies has not yet been addressed. Past human communities in volcanic areas have been repeatedly threatened by eruptive activity and subjected to short-term catastrophic events leading to major landscape changes^13^. It becomes crucial to consider the socio-ecological characteristics of the human societies, since their vulnerability or resilience relies on different aspects such as their settlement patterns, demography and socio-political organization and economic activities^25^. In fact, volcanism does not necessarily drive to disaster and societal collapses deterministically, as there are examples of resilient societies that coped with volcanic events^26^. In this context, the mobility of hunter–gatherers may have been an effective strategy to deal with the consequences of environmental constraints^27–29^. To understand past hunter–gatherer interaction with volcanic eruptions is important to consider post-eruption resilience measures but also the geological parameters of the eruption^30^.

**Figure 1:**
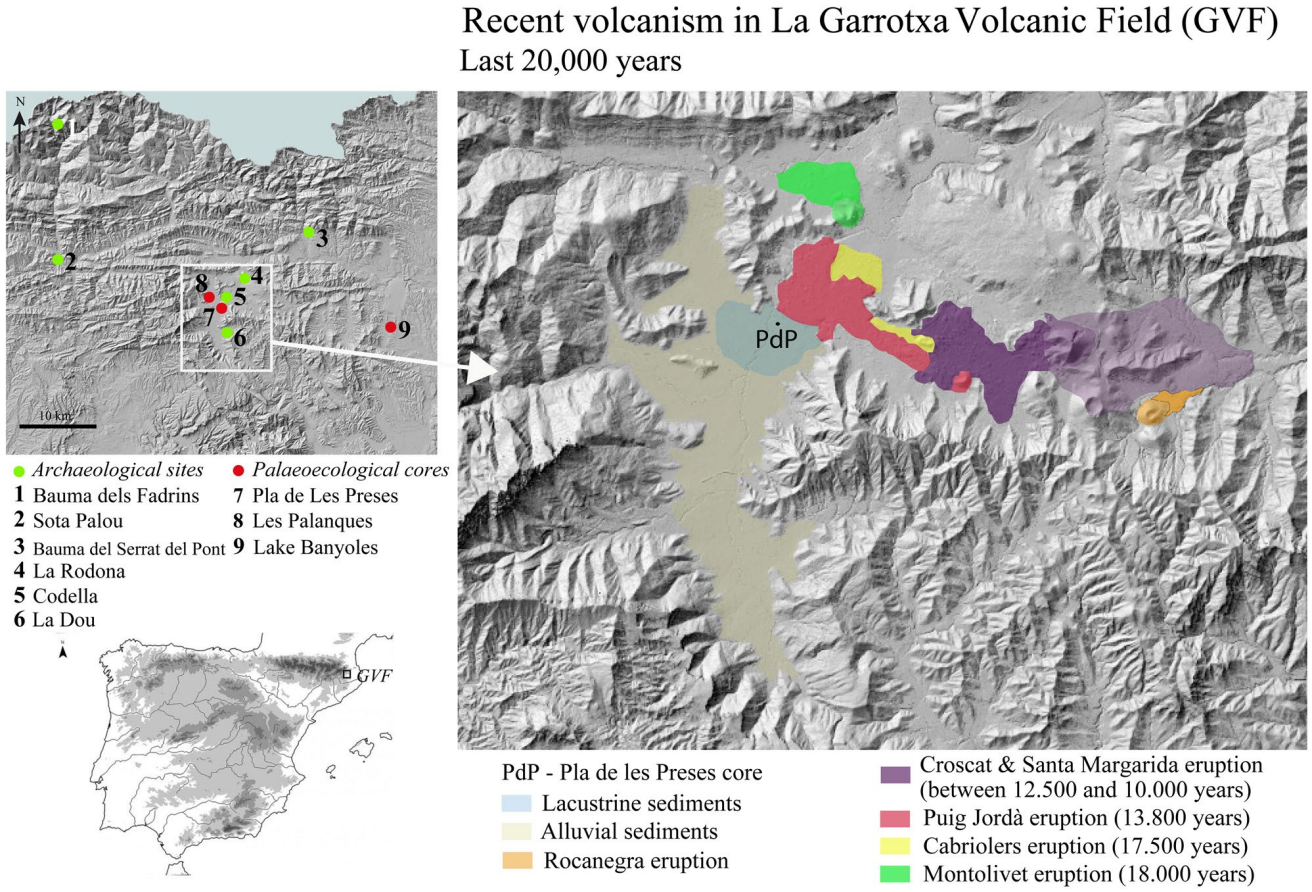
Location of La Garrotxa Volcanic Field in NE Iberia. Top left, archaeological sites and palaeoecological cores mentioned in the text are indicated. In the main map (right), lava flows and volcanos for the last 20,000 years are indicated. Map created with QGIS (https://www.qgis.org/es/site/), version 3.22, and modified in Adobe Illustrator version 23.0.5.

Successive lava flows in the La Garrotxa Volcanic Field dammed the Fluvià river and led to the formation of a lacustrine basin in the deepest part of the Vall d’en Bas valley (La Garrotxa, NE Iberia) (Fig. 1). The youngest lava flows were likely associated with the most recent eruptions from the Puig Jordà (17 ka BP^22^) and the Croscat (15.7–13.2 ka cal BP^31^) volcanoes. However, the ages of the eruptions are not well constrained as they have high uncertainty and low reliability. Recently, the Pla de les Preses sediment succession, at the Vall d’en Bas valley (Fig. 1) enabled precise radiocarbon dating of macrofossils and bulk sediment associated with tephra (Suppl. Files 1), identifying several eruptions in the period 14.0–8.4 ka cal BP, showing the potential of barrier-lake deposits to date volcanic eruptions. These lacustrine sediments also offered the possibility of conducting detailed palaeoenvironmental reconstructions involving regional landscape (vegetation) and local lacustrine environments (aquatic organisms). In that sense, the objective of this paper is to show the potential socio-ecological impact of monogenetic volcanism, integrating geochemical (XRF) and palaeobiological (pollen and non-pollen palynomorphs, sedimentary charcoal, ostracods, charophyte gyrogonites and diatoms) proxies and the archaeological record in the region, to assess how volcanism affected last hunter–gatherer communities and their surrounding environment.

## Results and discussion

### The last volcanic eruptions in NE Iberia

The monogenetic Quaternary La Garrotxa Volcanic Field^18,22^ forms part of the Catalan Volcanic Zone (north-east Iberian Peninsula)^32,33^, one of the alkaline volcanic provinces of the European rift system. It harbours more than 50 basaltic monogenetic cones that range in age from the Middle Pleistocene (*ca*. 350 ka BP) to the early Holocene and include cinder and scoria cones, lava flows, tuff rings and maars.

**Figure 2:**
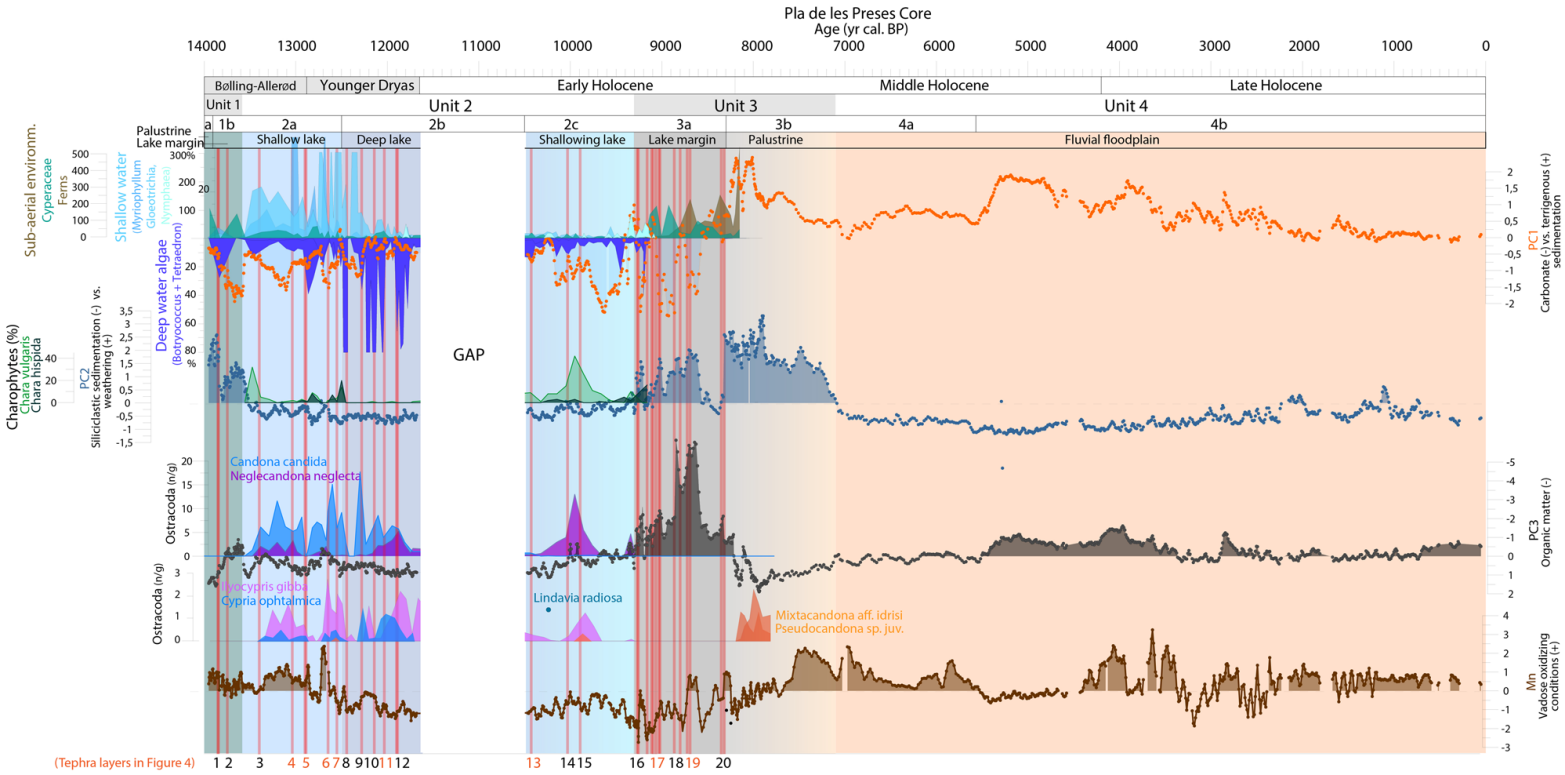
Paleoenvironmental evolution in Vall d’en Bas valley based on the main geochemical PCA and aquatic organisms (pollen, non-pollen palynomorphs, ostracods, gyrogonites and diatoms). The main environmental phases are indicated in colour frames (Green: palustrine before lake formation; Light blue: shallow lake; Dark blue: deep lake; Grey: lake margin; Grey to pale brown: palustrine; Orange: fluvial floodplain). The red bars indicate volcanic tephra layers.

The 15-m long sediment core from Pla de les Preses (Fig. 1) provides palaeoenvironmental data for the last 14,000 years (Suppl. Files 1), but we focus on the time interval 14–8 ka cal BP, since palaeobiological studies had richer results in lacustrine and wetland sediments in that period, and volcanic activity was not detected in fluvial sediments from 8.4 ka cal BP onwards (Suppl. Files 2). At the beginning of the sedimentary record, fluvial sedimentation was disrupted by coarse sands and volcanic tephra, linked with volcanic activity at *ca*. 14.0 ka cal BP. Lava flows from this eruption (by the Puig Jordà volcano, Fig. 1) would have built a barrier on the Fluvià river enabling the formation of a lake. The lacustrine environment lasted from 13.6 to 9.3 ka cal BP, thus spanning the time interval from the Late-Glacial (Bølling-Allerød and Younger Dryas) to the early Holocene. Several tephra layers (Suppl. Files 3) were deposited during a phase of more intense volcanic activity at 13.0–12.0 ka cal BP and another eruptive episode at 10.4 ka cal BP (Fig. 2). A shallowing process was accentuated by a cool and dry episode (Bond event 6)^34^, leading to the transition from lacustrine to palustrine conditions at 9.3 ka cal BP (Fig. 2). Tephra layers are also identified intercalated between these palustrine peaty layers, which correspond to the most recent eruptions at 9.4–8.4 ka cal BP (Figs. 2, 4). Around 8.2 ka cal BP, wetland areas converted to a fluvial floodplain again, suggesting a shallower water body and the incision of the volcanic dam by the river (Suppl. Files 2).

### Volcanic eruption induced wildfires and landscape changes

**Figure 3:**
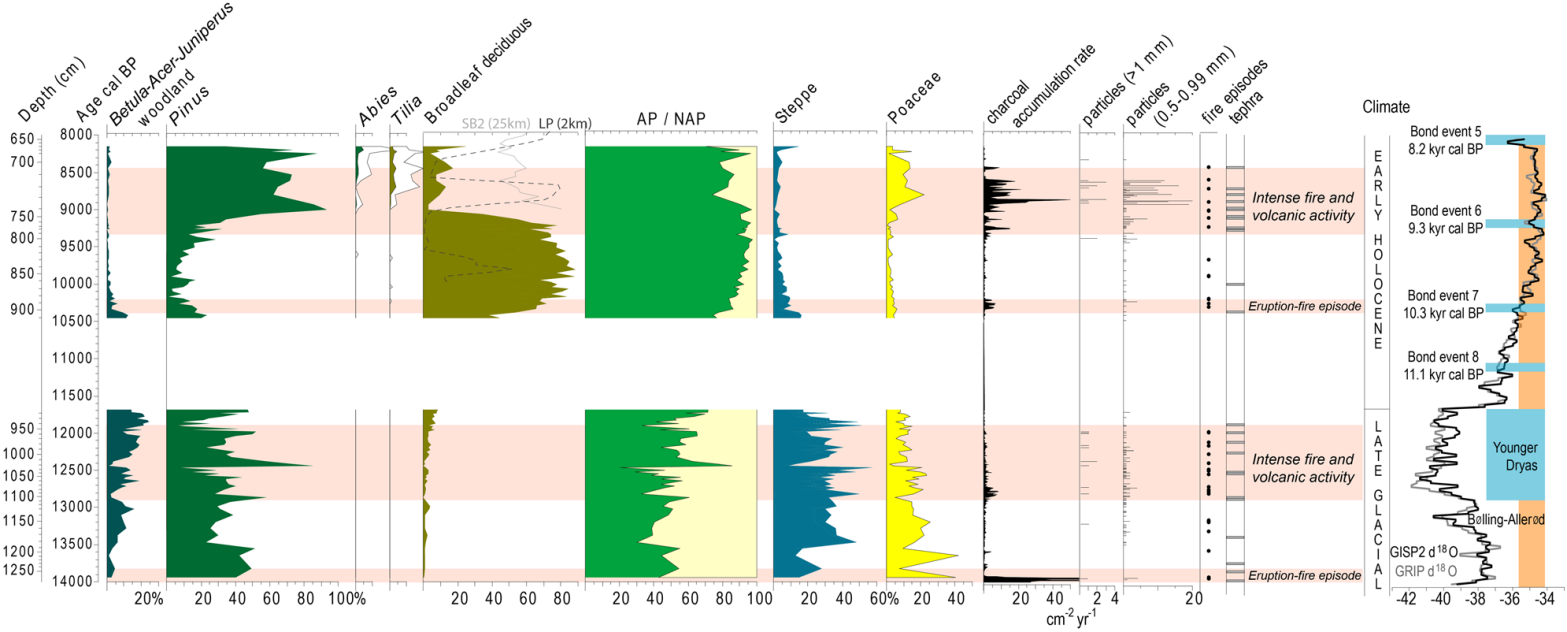
Percentage pollen diagram of selected taxa and categories. Data is plotted on an age scale (cal BP) and tephra layers were excised. Categories: broadleaf deciduous (*Quercus* deciduous, *Corylus, Ulmus, Fagus*), steppe (*Helianthemum*-t, *Sanguisorba*, *Artemisia*, Amaranthaceae, *Plantago*, Apiaceae, *Rumex*, *Galium*-t, *Filipendula*). In the broadleaf deciduous curve, the discontinuous line shows values in Les Palanques core (2 km distance from PdP) and the grey line shows SB2 Banyoles core (25 km away). Sedimentary charcoal data is plotted as accumulation rate of charcoal particles, *n* of particles > 1 mm width and 0.5–0.99 mm width, and charcoal peaks identified by numerical analyses. Red shadings indicate periods of intense eruptive and fire activity, grey lines show events of tephra deposition. Greenland isotopic curves (GRIP and GISP2)^42,43^ are plotted and cooling episodes/cold phases (blue) and warmer periods (orange) are indicated.

**Figure 4:**
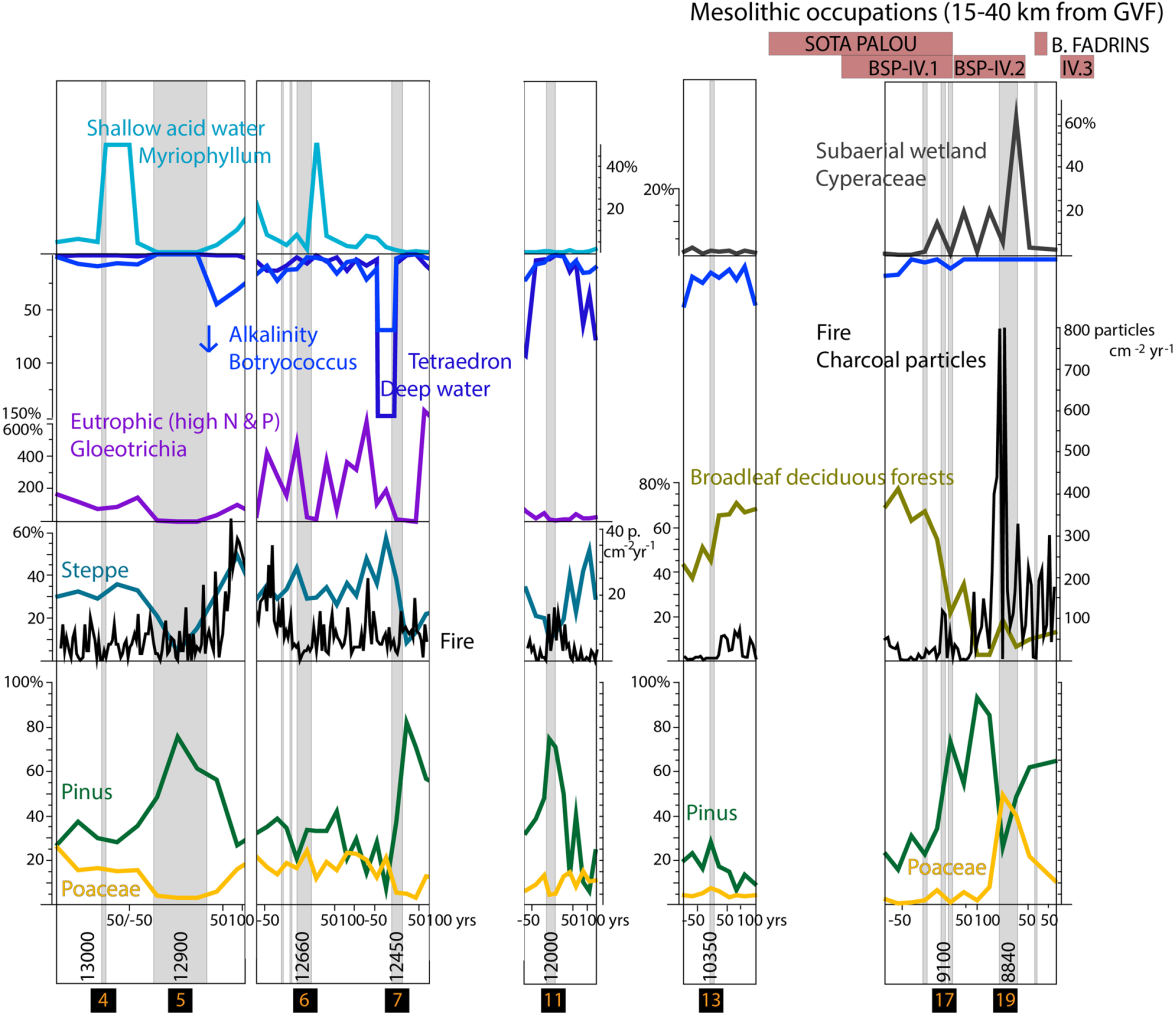
Multi-proxy diagram showing environmental shifts in relation to selected tephra. Data is plotted on a depth scale (cm) and grey shadings indicate tephra deposition events. Occupation layers from Late Mesolithic sites in the region (15–40 km from the La Garrotxa Volcanic Field) are shown in the top-right corner: Sota Palou (10,200–9100 cal BP), Bauma del Serrat del Pont IV.1 (9400–9100 cal BP), IV.2 (9100–8800 cal BP), IV.3 (8600–8400 cal BP), Bauma dels Fadrins (8700 cal BP).

Pollen analysis provided data about vegetation history from 14.0 to 8.0 ka cal BP. During the Late-Glacial, the prevailing landscape ecosystems were steppes and grasslands (40–60%) and forests were dominated by *Pinus* and woodlands of *Betula*, *Acer* and *Juniperus*. This characteristic landscape is consistent with cool climate conditions during the Late-Glacial. However, the transition from the warmer Bølling-Allerød to the colder Younger Dryas period did not cause substantial change of vegetation (Fig. 3), confirming a milder Younger Dryas in NE Iberia^35^, as attested previously in the Central Pyrenean region^36–38^. After the first evidence of links between eruption and fire episodes in 14.0 and 13.5 ka cal BP, the major Late-Glacial phase of intense fire and volcanic activity occurred during 13.0–12.0 ka cal BP (Fig. 3), confirming the significant role of volcanic activity in initiating fire episodes^39^, among other factors such as dry Late-Glacial climatic conditions, as attested in other Iberian records^37,40^. Volcanic eruptions and subsequent forest fires caused short-term episodes of expansion of pine forest and decline of Late-Glacial woodlands (*Betula*-*Acer*-*Juniperus,* Fig. 3) and steppes. However, the Late-Glacial vegetation was not disrupted dramatically, showing recovery during the following 50–100 years after the eruptions (Fig. 4).

The *Pinus* and *Betula*-*Acer*-*Juniperus* dominated Late-Glacial woodlands, as well as the grasslands and steppes, were replaced by broadleaf deciduous forests (mainly *Quercus* and *Corylus*) due to a warmer and wetter early Holocene climate between 11.7 and 10.5 ka cal BP^41,42^ (Fig. 3). This rapid expansion of broadleaf deciduous forest, which reached maximum values around 10.3–9.2 ka cal BP (Fig. 3) was interrupted by volcanic activity, combined with the cooling Bond Event 6 (9.3 ka cal BP). The first evidence of early Holocene volcanism is observed at 10.4–10.3 ka cal BP, provoking fire episodes and a limited impact on deciduous woodlands (see tephra 13 in Fig. 4). Later, at 9.4–8.3 ka cal BP the broadleaf deciduous forests collapsed in conjunction with more frequent fire episodes and volcanic eruptions. This suggests that fire episodes induced by volcanic eruptions dramatically impacted on broadleaf deciduous forests, the predominant vegetation in this region during the early and middle Holocene^43^, triggering the expansion of grasslands and of secondary *Pinus*-dominated woodlands. Although palaeoecological evidence attested landscape transformation by the use of fire by last hunter–gatherer communities in Western and Central Europe^44–46^, the characteristic sparse Mesolithic populations in NE Iberia^43^ would have had a limited impact on the landscape. The detailed records for eruptions at 9.15–9.1 ka cal BP and 8.84–8.77 ka cal BP (tephra 17 and 19 in Fig. 4) show major impacts of eruptions and wildfires in a two-step process: firstly, dramatic impact on broadleaf deciduous forests, followed by pine woodlands expansion; secondly, the burning of pine forest and the expansion of clearings dominated by grasslands (Poaceae) (Fig. 4) and a moderate expansion of disturbance-sensitive trees (*Abies* and *Tilia*^47^) when the frequency of volcanic eruptions decreased and climatic conditions became warmer (Fig. 3). Recurrent eruptive activity and more intense wildfires prevented deciduous forests to rapidly recover. Nevertheless, a previous study in the Vall d’en Bas valley (Les Palanques, 2 km to the west, Fig. 1) showed that broadleaf deciduous forests declined in conjunction with peaks in *Pinus* and Poaceae at 9.5–9.0 and 8.5–8.3 ka cal BP and recovered from 8.1 ka cal BP onwards^43,48^ (Fig. 2), pointing to a significant impact of volcanic activity on vegetation at the local scale and slower recovery processes during the early Holocene than during the Late-Glacial. Likewise, an expansion of *Pinus* and Poaceae and decline in broadleaf deciduous forests is documented around 8.8–8.6 ka cal BP (Fig. 2) at extra-local scale, 15 km away, in Bauma del Serrat del Pont (highest values of *Pinus* and Poaceae in Mesolithic layers^49,50^), and 25 km away, in Lake Banyoles^51^ (Fig. 1). However, the pollen record from Lake Banyoles clearly shows maxima of broadleaf deciduous vegetation in conjunction with low fire activity during the period 9.0–7.5 ka cal BP^51^, when climatic conditions were wetter and warmer (Holocene Climate Optimum). Overall, the evidence of dramatic environmental changes from the Pla de les Preses records support the view that local factors (e.g. volcanic activity) rather than large-scale factors such as climate drove vegetation and fire dynamics in the Vall d’en Bas valley. Cooling episodes during the Younger Dryas and the Holocene (10.3 and 9.3 kyr cal BP) acted as amplifiers, configuring drier landscapes that are more prone to wildfire occurrence and spread.

### Volcanism and limnology, ecological impact in aquatic organisms

The multi-proxy approach developed in this work clearly evidenced the impact of volcanic eruptions on limnological conditions, as shown by the abrupt declines in aquatic plants (genus *Myriophyllum* in the Late-Glacial, genus *Nymphaea* in the early Holocene), algae (*Botryococcus*, *Tetraedron*, charophytes, diatoms), cyanobacteria and ostracods (*Candona candida*, *Ilyocypris gibba*, *Neglecandona neglecta*) after eruptive episodes (Fig. 2). During the Late-Glacial, the deposition of volcanic tephra, charcoal and ash from wildfires altered water conditions, resulting in lower alkalinity, as inferred by the predominance of the acid-resistant algae *Botryococcus*^52^ in deep water phases and the predominance of the aquatic plant *Myriophyllum* in shallower phases (tephras 5 & 7 and 4 & 6, respectively, Fig. 4). Limnological changes imposed by volcanism limited life on aquatic organisms in the short term, but *Gloeotrichia* played a significant role as pioneer in phases of poor nutrient availability^53^, spreading fast perhaps due to fixing nitrogen and the high availability of phosphorus. These conditions were also favourable for *Myriophyllum*, a macrophyte growing in waters with high nitrogen content and phosphorus availability^54^ and that prefers acidic waters^55^ (shallow lake phase in Unit 2a, Fig. 2). Peaks in cyanobacteria and Chlorophyta show how the lake trophism changed from oligotrophic, immediately after volcanic eruptions, to eutrophic in periods of 50 to 100 years (Tephra 5, 6, 7 in Fig. 4). Similarly, the planktonic diatom *Lindavia radiosa* was only observed after the volcanic eruption at 10.24 ka cal BP, which indicates an increase in the lake trophic state and the presence of a water column. However, the most abundant benthic diatoms in the pre-tephra sample (10.42 ka cal BP) were still abundant after the volcanic eruption sample (10.24 ka cal BP), despite the scarcity of diatoms observed in the tephra layer (Suppl. Mat. 2). These results indicate a rapid recovery of diatom assemblages and other aquatic organisms after the 10.35 ka cal BP eruption (Fig. 2).

Therefore, volcanism enhanced water acidity and aquatic organisms adapted to these new conditions. However, these disturbance events were followed by fast recovery processes, especially during the Late-Glacial, when the deeper lake showed high resilience. Nevertheless, during the early Holocene, the combination of a shallowing process enhanced by climate change, volcanic activity and high-intensity local wildfires affected local aquatic communities, which did not recover after the eruptive activity at 9.3–8.8 ka cal BP (Figs. 2, 4).

### Volcanism and human resilience during the Late Mesolithic in NE Iberia

The archaeological record shows a gap of human settlement in the GVF area since the Upper Palaeolithic (La Rodona, Olot, *ca*. 33–24 ka cal BP^56^) and human communities did not settle again until the Neolithic (Codella and La Dou, 6.7 ka cal BP^57,58^), suggesting that this area was hostile for the last hunter–gatherers during the Late-Glacial and early Holocene. In this context, it is worth mentioning that the low Mesolithic population density is not exclusive to the GVF area. This period has not attracted especial attention in archaeological research in the NE Iberian Peninsula given the scarcity of archaeological records with evidence of occupations during the early Holocene, as opposed to neighbouring areas (Ebro basin, eastern coast of Iberia and Mediterranean coastal area of France)^59^. The scarcity of archaeological evidence in this region of NE Iberia during the Late-Glacial and the early Holocene is probably not driven by climate, as the archaeologically inferred gap of occupations is not restricted to colder phases (e.g. the Younger Dryas). Instead, the occupation gap could have been driven by settlement patterns or post-depositional processes affecting the preservation of archaeological sites. Mesolithic occupations are documented 15 km away from the GVF, in the Llierca valley (Fig. 1). There, the rock shelter site Bauma del Serrat del Pont (BSP) (Fig. 1) shows 4 layers of Mesolithic occupations, from 9.4 to 8.0 ka cal BP (9.45–9.1, 9.1–8.8, 8.6–8.4, 8.3–8.0 ka cal BP)^60^, contemporary to the most frequent early Holocene volcanic eruptions in the GVF area. These communities were located in ecotones providing diverse resources for their terrestrial foraging economies, including many wild hunted species such as red deer, roe deer, wild boar and ibex^61^ and plants and fruits such as acorns, sorb apples, hazelnuts and strawberry tree fruits^62^. Overall, the coincidence of eruptive episodes and changes in archaeological layers (Fig. 4) suggests that hunter–gatherer communities at BSP abandoned temporarily the site at 9.1 (transition from layers IV.1 to IV.2) and 8.8 ka cal BP (200 years of gap between layers IV.2 and IV.3) in response to enhanced volcanic activity. Different hazard agents likely acted in the proximal impact zone (up to at least 50 km), including lava flows, tephra deposition, ash storms, gases, aerosols, pyroclastic flows and earthquakes^30^, affecting flora and fauna (bioresources) as well as the quality of air and water. In that context, other Mesolithic sites were occupied during this period of environmental disturbance in the region, including Sota Palou (10.2–9.1 ka cal BP^63^) and Bauma dels Fadrins (8.7 ka cal BP^64^) (28 and 38 km from the GVF, respectively), suggesting a spatially restricted impact of the last volcanic eruptions in GVF. BSP was re-settled at 8.6–8.4 and 8.3–8.0 ka cal BP, suggesting that the overall social system was not challenged, and Mesolithic communities did not collapse. Hunter–gatherer societies dwelling in the proximal impact zone may have abandoned the area temporarily during high volcanic activity periods, but returned afterwards, proving high reorganization capacity. Thus, the low magnitude monogenetic volcanic eruptions in GVF did not have such a dramatic effect as larger magnitude explosive volcanic eruptions causing the collapse on past hunter–gatherer communities^30^. What emerges from the analysis of the archaeological and palaeoecological records in the La Garrotxa region is that the last hunter–gatherer communities were resilient at an extra-local scale (15 to 40 km from the GVF) against the local-scale impact of monogenetic volcanism, which indeed affected the settlement in the Vall d’en Bas valley since the Upper Palaeolithic to the early Neolithic (gap of human occupations in 24–6.7 ka cal BP at local scale). Their flexible nomadic strategies and foraging economies were likely an efficient source of risk management to face episodes of vulnerability caused by volcanic eruptions. Mobility was the risk mitigation strategy enabling the resilience of past hunter–gatherers in NE Iberia against volcanic eruptions, as there is no clear evidence in the archaeological record of other possible crisis management strategies such as storage, exchange, diversification or intensification^30^.

## Concluding remarks

**Figure 5:**
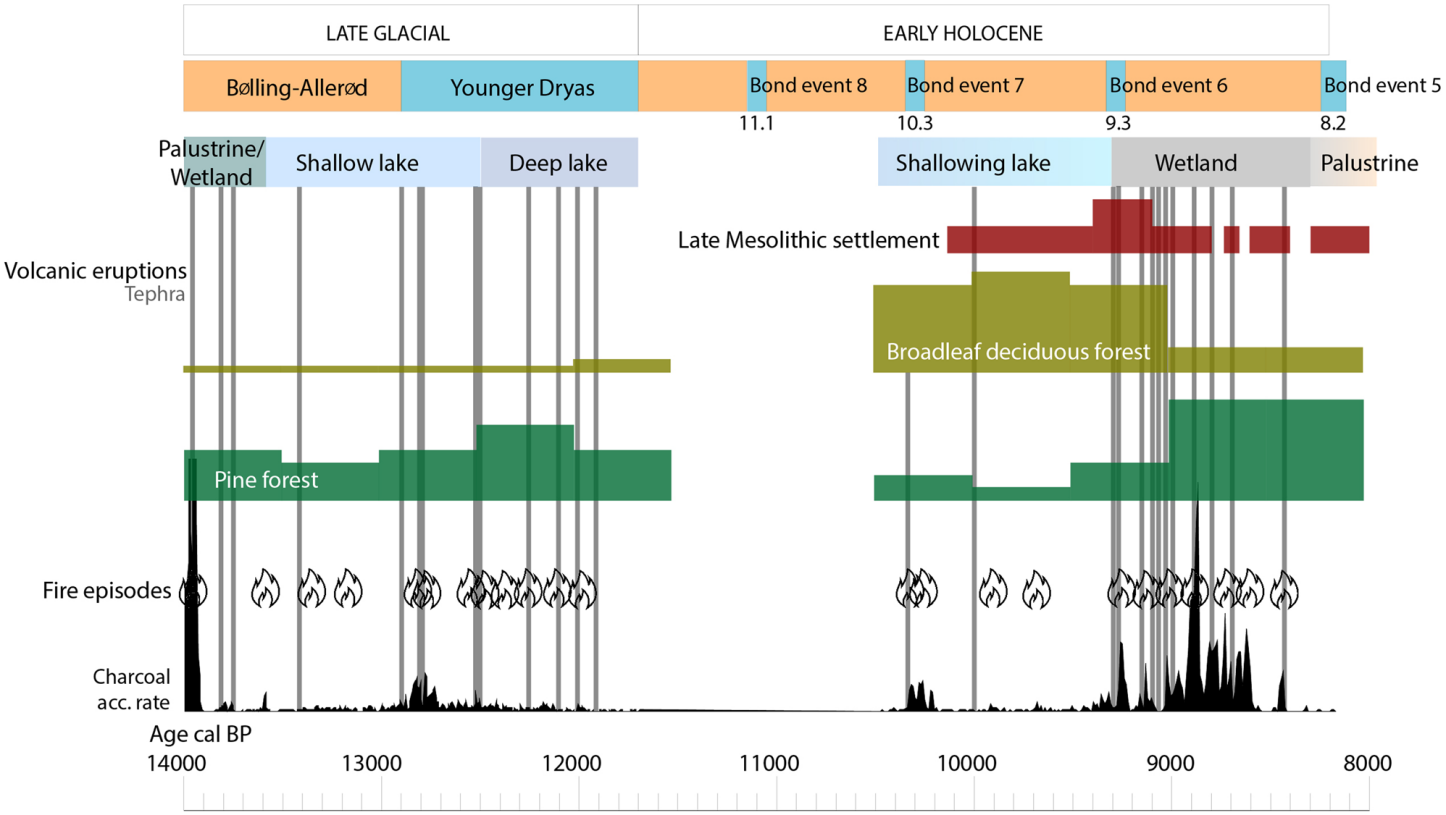
Synthetic diagram including climate change events, sedimentary evolution, volcanic eruptions (tephra), fire episodes, charcoal accumulation rate, vegetation dynamics and human settlement during Late Mesolithic in the region.

This work provides insights on how environmental disturbance by monogenetic volcanism influenced geomorphology, vegetation, aquatic organisms and past human societies. New Late-Glacial-early Holocene volcanic eruptions, not previously reported in GVF, are presented, and their volcanic stratigraphy and age are constrained, reconstructing their major palaeoenvironmental impacts (Fig. 5). Volcanic activity had a significant environmental impact producing intense fires and causing dramatic changes in landscape vegetation on a local scale. Volcanism initiated intense wildfire episodes affecting woodlands (*Acer-Betula-Juniperus* in the Late-Glacial; *Corylus-Quercus* deciduous in the early Holocene), except for pine forests, which expanded colonising the disturbed areas. This study shows that Late-Glacial cold steppes recovered quicker from volcanic disturbance than the Holocene broadleaf deciduous forests. In addition, the records indicate that tephra deposits also altered the lake ecosystem, enhancing water acidity and boosting the aquatic organisms adapted to these conditions. These disturbance events were followed by fast recovery at decadal to centennial scales, especially during the Late-Glacial, when the deeper lake showed higher resilience.

This study enables a better understanding of palaeoenvironmental dynamics and ecological changes that occurred during the youngest volcanic eruptions in GVF. Volcanism had a large socio-ecological impact at local scale (radius < 15 km) causing intense fire activity and remarkable changes in vegetation (forests), aquatic communities (lake) and a gap of human settlements until the Neolithic (6.7 ka cal BP). Most importantly, the new records add important evidence to explain the Late-Mesolithic population resilience against volcanism at an extra-local scale, using mobility as a source to face vulnerability episodes, since hunter–gatherer groups abandoned (during high volcanic activity periods) and re-occupied (during volcanic quiescence) the BSP site four times during the period 9.4–8.0 ka cal BP. Thereby, this study proves the socio-ecological impact and the interest in developing interdisciplinary palaeoecological research to address local-scale landscape transformations caused by the most frequent volcanism on Earth, monogenetic volcanism.

## Methods

### Coring

A 15-m long core was obtained using a mechanical rotary drilling machine (TP-50/D) from the lowest part of the valley of La Vall d’en Bas (Girona, Spain) (UTM 455189.0 X/4667356.0 Y/458.1 m asl), in the area known as Pla de les Preses (PdP).

### Dating and depth-age model

The depth-age model was established with RBacon^65^ and uses 15 control points. Thirteen of these points are based on radiocarbon (^14^C) dates measured on bulk sediments and two points are based on ^14^C dates measured on terrestrial plant remains (*Pinus* sp. needles and seeds) (Suppl. Files 1). As the latter ^14^C dates very likely provide more accurate sediment-deposition ages^66^, narrower student‐t error distributions were applied were applied for plant-macrofossil dates. Abrupt sedimentation events (e.g. tephra deposits) were excised, and a sedimentation hiatus was set at 945 cm depth with a maximum hiatus length of 1500 years. The hiatus, which was probably caused by poor sedimentation recovery during the coring, led to the absence of sediments for the period between 11.7 and 10.5 ka cal BP.

### Stratigraphy and sedimentology

The lithostratigraphic study of the core was defined taking into account the different sedimentary facies (Suppl. Files 2). Sedimentary facies were defined by visual macroscopic description and microscopic observation of smear slides following LRC procedures^67^ (and by mineralogical, organic and geochemical compositions). Different stratigraphic units were defined along the core and their depositional environments and processes inferred based on their sedimentological characteristics^68^.

### Geochemistry

A high-resolution geochemical analysis (1 cm step size) of the core was performed using an Avaatech XRF Core-scanner at the Corelab Laboratory (University of Barcelona). The analysis was performed using a Rhodium source under two different working conditions: 1) with an X-ray current of 800 μA, at 10 s count time and 10 kV x-ray voltage for the measurement of Al, Si, P, S, Cl, Ar, K, Ca, Ti, V, Rh, Cr, Mn and Fe; 2) with an X-ray current of 2000 μA, at 25 s count time, 30 kV x-ray voltage and using a Pd filter, for the measurement of Ni, Cu, Zn, Ga, Ge, As, Br, Rb, Sr, Y, Zr, Nb and Pb. This method allowed a semi-quantitative analysis of the elemental chemical composition from Al to U, based on the proportion of counts per second (cps) for each element compared to the rest. The most abundant and significant elements (Al, Si, Cl, K, Ca, Ti, V, Mn, Fe, Ni, Cu, Zn, Ga, Br, Rb, Sr and Pb) were selected for multivariant statistical analysis (PCA) to reduce the number of variables and define the main phases and processes involved in the formation of the core record according to their chemostratigraphy. Before the PCA analysis all unreliable measurements were removed, so as not to obscure the statistical treatment of the data. XRF geochemical data was normalized using centred log-ratio transformation^69,70^ using CoDaPack software^71^ and processed with multivariate statistics. A Principal Component Analysis was performed using SPSS 23.0 software at correlation mode, factor scores were calculated, and rotated (Varimax) and not rotated solutions were evaluated and the most suitable to geochemical data variance selected (Suppl. Files 2).

### Pollen and non-pollen palynomorphs

Pollen samples were obtained each 3–5 cm in organic clayish and peaty facies and each 10 cm in inorganic silts in the fluvial layers. Samples were processed following standard methods^72^ including treatment with HCl and NaOH, flotation in Thoulet heavy liquid, treatment in HF, and finally mounting in glycerine. At least 300 pollen grains of terrestrial taxa were counted using an Olympus Bx43 microscope fitted with 10× oculars and 40/60× objectives. Hygrophytic plants (Cyperaceae, Ranunculaceae *Typha latifolia* and *Typha/Sparganium*) and aquatic plants (*Myriophyllum, Nuphar, Nymphaea, Potamogeton*) were excluded from the pollen sum. Pollen grains were identified using a pollen atlas^73^. The identification of non-pollen palynomorphs (NPPs) followed van Geel^74^, van Geel et al.^75^, Revelles et al.^76^ and Revelles and van Geel^77^. Pollen percentages were calculated with respect to the pollen sum and diagrams plotted using Tilia software^78^. Despite pollen analysis was applied to the whole succession, some samples were poor in pollen for the top 650 cm, and data is only provided for the bottom part of the core (14.0–8.0 ka cal BP).

### Sedimentary charcoal and plant macrofossils

Quantification of charcoal particles was performed with the sieving method^79^ with a 150 μm mesh size^80^ in order to identify fire episodes. Samples of 1 cm^3^ were retrieved each 1 cm from the whole sediment sequence. Samples were first soaked in 10% H_2_O_2_ for 12 h to deflocculate and bleach the sediment and then sieved on a 150 μm sieve under a soft-water jet. Very organic samples were additionally soaked in 10% NaOCl for 4 h to further bleach the organic material. The bleached sieving residue was analysed under a stereoscopic microscope (Leica M80 at 60x) equipped with a camera CMEX DC 5000 connected to a computer with an image-analysis software (WinSeedle, Regent Instruments Canada, Inc.) that allowed the measurement of charcoal concentration, charcoal areas of individual particles and the cumulative sum of charcoal-particle areas^81^. The CharAnalysis software^82^ was used to calculate charcoal accumulation rates (charcoal cm^−2^ yr^−1^) and to detect fire episodes. The analysis was run into two time-windows, one for Holocene samples (8.2–10.5 ka cal BP), one for Late Glacial ones (11.7–14.0 ka cal BP). Charcoal counts, sample volume and sample depths were interpolated to a constant temporal resolution of 10/6 (Holocene/Late Glacial) years before calculating charcoal accumulation rate to account for unequal sampling intervals resulting from variable sediment accumulation rates. No data transformation was performed before applying numerical analysis. The slowly varying mean or background component (Cback) was modelled through a LOWESS function with two different smoothing windows (400 yrs in Holocene, 300 yrs in Late Glacial) based on the highest values of goodness of fit test. Finally, two categories of charcoal particles width were plotted (Fig. 3) to explore the local signal of fires.: > 1 mm width and 0.5–0.99 mm width.

Some macrofossils were recovered within sedimentary charcoal samples. Despite organic macrofossils were affected by H_2_O_2_ processing, most seeds could be identified using a stereoscopic microscope (Leica M80 at 60x). Identifications were made based on literature^83,84^ and the reference collection of seeds at the University of Montpellier).

### Ostracods and charophyte gyrogonites

Samples of around 20 g of sediment were retrieved for ostracods and charophyte analyses every 10 cm in the peaty and lacustrine facies (600–1470 cm). The samples were rinsed in water (with use of H_2_O_2_ to disaggregate clayish samples) and sieved through 250 μm. Finally, the samples were dried and all ostracod remains and charophyte gyrogonites were picked up with a fine brush.

All ostracod remains (shells and disarticulated valves) were identified to species level whenever possible, following mainly Meisch^85^ and Fuhrmann^86^. Densities were estimated as the number of valves per gram of dry sediment. The qualitative taxonomic characteristics used to identify the charophyte gyrogonites were: apical zone, basal structures (presence or absence of a basal column, shape of the basal plate) as well as other features such as overall outline and number of spiral turns (or ridges) visible in lateral view^87^. The observation and measurements were made with a stereomicroscope at 400x. Length was measured as the longest polar axis (LPA = vertical axis); width as the largest equatorial diameter (LED = horizontal axis at the largest diameter). The length/width ratio was also calculated and expressed as the isopolarity index (ISI = LPA/LED × 100).

### Diatoms

Twelve samples were selected for diatoms analysis before and after 4 tephra layers to assess the impact and the recovery of the diatoms community to tephra deposition in the lake ecosystem. All these samples were treated with 33% hydrogen peroxide (H_2_O_2_) and HCL (1 M). Subsequently samples were mounted in Naphrax (R.I. = 1.7) following the method described in Battarbee et al.^88^. Diatom identification was followed mainly Krammer and Lange-Bertalot^89–92^, but the diatoms nomenclature (basionym) were updated to accepted names following currently accepted nomenclature^93^. Unfortunately, diatom preservation was sufficient only in two Holocene samples (before and after T6) to identify and count enough, at least 10 diatom valves per slide. Diatom dissolution, and therefore their absence in sediment records, could result from an increase in lake salinity or alkalinity^94^.

## Supplementary Information

Supplementary Information 1.

Supplementary Information 2.

Supplementary Information 3.

## Data Availability

The datasets generated during the current study are available in the Neotoma Palaeoecological Database repository (https://data.neotomadb.org/56691).
