# Supplementary material for: Socio-ecological impact of monogenetic volcanism in the La Garrotxa Volcanic Field (NE Iberia)
Source: Sci Rep. 2023 May 20;13:8168. doi: 10.1038/s41598-023-35072-0 (PMC10199944; doi:10.1038/s41598-023-35072-0)
Supplement: Supplementary file 1 — Supplementary Information 1. [file 41598_2023_35072_MOESM1_ESM.docx]

**SUPPLEMENTARY FILES 1**

**The Pla de les Preses succession. Dating and age-depth model.**

The 15 radiocarbon dates obtained from bulk sediment and *Pinus* sp. macrofossils yielded positive results (Table S1). The dated samples constrain the core record between 14024-2057 cal. yr BP (Table S1). Nevertheless, the age-depth model calculated through rBacon allowed to calculate the age of the entire core ranging from present to ca.14 ka cal. BP, covering the Late Glacial to the Late Holocene (Fig. S1).

| Sample name | Lab. code | Depth (cm) | Sample type | AMS Radiocarbon Age (BP) | Cal. yr BP (2σ) |
| --- | --- | --- | --- | --- | --- |
| Surface |  | 0 |  |  | -45 to -55 |
| PdP-2/11 | Beta-507261 | 191 | Bulk sediment | 2160±30 | 2306-2057 |
| PdP-4/48 | Beta-507262 | 494 | Bulk sediment | 4820±30 | 5604-5475 |
| PdP-5/09 | Beta-411093 | 606 | Bulk sediment | 7100±30 | 7995-7855 |
| PdP-5/95 | Beta-406774 | 692 | Bulk sediment | 7340±30 | 8274-8034 |
| PdP-6/30 | Poz-119262 | 766 | Bulk sediment | 8180±40 | 9259-9022 |
| PdP-6/39 | Poz-119260 | 775 | Bulk sediment | 8230±50 | 9398-9030 |
| PdP-6/86 | Beta-406775 | 822 | Bulk sediment | 8260±30 | 9399-9129 |
| PdP-7/53 | Poz-83394 | 939 | *Pinus* sp. needles | 9210±80 | 10567-10235 |
| PdP-7/62 | Poz-119261 | 948 | Bulk sediment | 11560±60 | 13531-13272 |
| PdP-7/75 | Beta-411094 | 961 | Bulk sediment | 10950±40 | 12871-12711 |
| PdP-8/74 | Beta-411095 | 1084 | Bulk sediment | 10940±40 | 12902-12708 |
| PdP-8/123 | Poz-83395 | 1133 | *Pinus* sp., seed | 10721±60 | 12729-12573 |
| PdP-9/73 | Poz-83396 | 1229 | Bulk sediment | 11060±60 | 13067-12779 |
| PdP-9/137 | Beta-406776 | 1293 | Bulk sediment | 12240±40 | 14293-14001 |
| PdP-11/13 | Beta-411096 | 1447 | Bulk sediment | 12040±40 | 14024-13762 |

**Supplementary table 1.1***.* Radiocarbon dated samples from Pla de les Preses core. Reported 16 control points were used to constrain the depth-age model.

We set a hiatus at 945 cm depth, where a transition between two sediment drives coincides with an abrupt pollen-assemblage change that suggests the occurrence of a sediment gap at that depth; the occurrence of a hiatus in sedimentation is not supported by sedimentological or climatic indicators and seems to be caused by a sediment loss during coring operation. Finally, the sedimentary sequence includes several volcanic tephra layers that were likely deposited in a very short period of time (days). These layers representing abrupt events of sedimentation were excised to perform the age-depth model (vertical grey bands in Fig. S1).


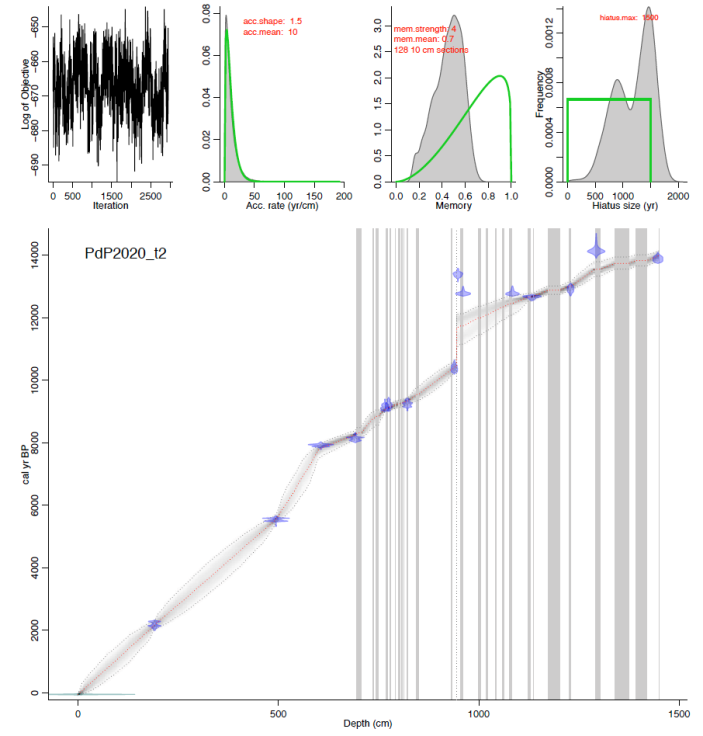


**Supplementary figure 1.1***.* Depth-age model of PdP core with student-t error distributions. Note the excised abrupt sedimentation volcanic tephra layers (grey vertical bands), and a hiatus at 945 cm depth
