# Supplementary material for: Socio-ecological impact of monogenetic volcanism in the La Garrotxa Volcanic Field (NE Iberia)
Source: Sci Rep. 2023 May 20;13:8168. doi: 10.1038/s41598-023-35072-0 (PMC10199944; doi:10.1038/s41598-023-35072-0)
Supplement: Supplementary file 2 — Supplementary Information 2. [file 41598_2023_35072_MOESM2_ESM.docx]

**SUPPLEMENTARY FILES 2**

**1. The Pla de les Preses succession. Stratigraphy and Sedimentology.**

Four main lithostratigraphic units (U1 to U4) were defined in the core including 8 different sedimentary facies (Fig. S1). In the basal part of the core, U1 ranges from the bottom until 1300 cm depth. It includes orange to greyish carbonate mud and centimetre-scale fine sand layer alternation (Facies G) that evolves to massive grey carbonate mud (Facies B) at 1412 cm and is present until 1380 cm depth. The grey carbonate mud includes a 5-centimetre-wide black peaty organic layer with vegetal remains (Facies C) at the bottom and gradually becomes sandier upwards. Between 1380 cm and 1305 cm depth very coarse to gravelly siliciclastic sands (Facies D) with abundant organic mud clasts and bioclasts are present in two 20 and 33-centimetre-thick sand layers separated by a 20-centimetre-thick siliciclastic grey to black organic mud layer (Fig. S1).The siliciclastic mud between both sand layers and another two millimetre to centimetre scale whitish to grey clay layers with sharp contacts at 1400 cm and 1350 cm depth are significantly enriched in Zr and Al (also Mn and Fe) indicating a possible content of volcanic sediment (Facies E) (Figs. S1, S2).

From 1305 to 815 cm depth, U2 is mainly composed of massive grey carbonate mud with some carbonate bioclast-rich intervals (Facies B) (Fig. S1). It includes 15 intercalations of 5 millimetre to 25 centimetre-thick Zr-rich fine black sand (Facies D) and/or whitish to reddish grey laminated clay layers (Facies E) similar to the observed before in U1. Soft sediment deformation structures are visible in some of these layers, mainly when they include basal sand layers that form flame structures (Fig. S1).

U3 (815 to 570 cm depth) is mainly composed of an alternation of black organic carbonate to peaty mud (Facies C) that includes abundant centimetre- to decametre-scale bioclastic carbonate mud layers, and millimetre- to centimetre-scale whitish grey siliciclastic clay laminae (Facies E) (Fig. S1). Fine lamination is sometimes disturbed due to soft sediment deformation features and possible root bioturbation traces. At the top of U3 the massive organic mud becomes less organic and more siliciclastic (Facies F), rapidly changing to massive greyish mud with fine sandstone intercalations.

Finally, from 570 cm depth until the top, U4 is mainly composed of brownish orange siliciclastic sandy mud composed of an alternation of centimetre-scale fine sand and clay laminas (Facies F) (Fig. S3). Facies F present abundant sand-rich intervals of brown fine sands with mud laminations (Facies G), in the upper 3 meters of the core, intercalated in green to brownish orange massive siliciclastic mud intervals (Facies H) between 500 to 250 cm depth (Figs. S1, S2). Some scattered millimetre-scale charcoal fragments are present.

Supplementary figure 2.1. PdP core stratigraphy and main lithofacies differentiated along the PdP Core.


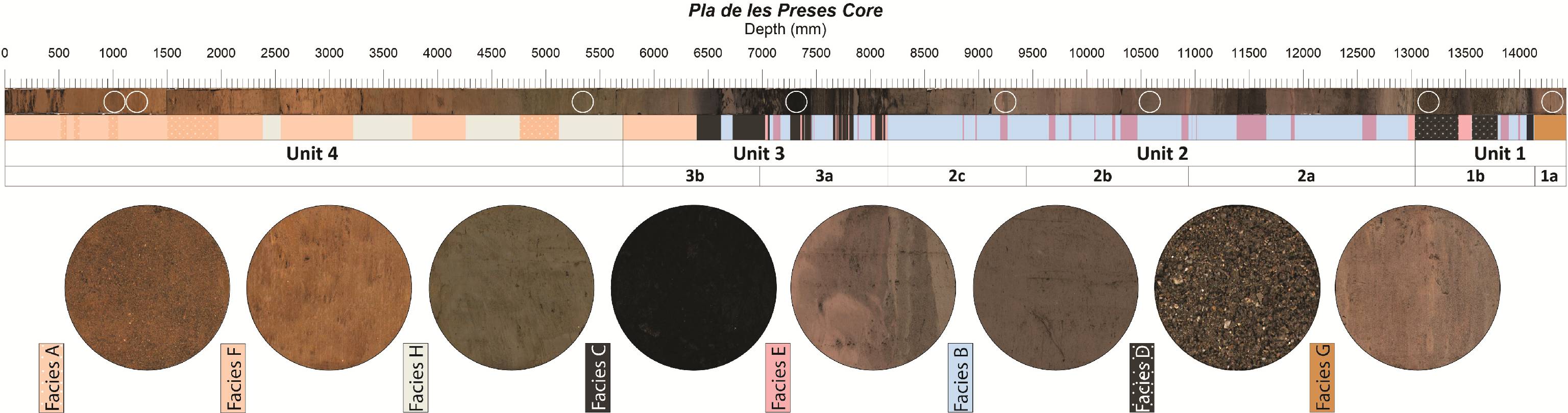


**2. The Pla de les Preses succession. Geochemical analysis**

The described lithostratigraphic units consist of geochemically well differentiated sediment types (Fig. S2): mixed carbonate mud (Ca-rich) (U1, U2 and U3), sometimes with relatively high organic content (high Cu, Ni, Br presence) (U2), siliciclastic (Al, Si, K) fine-grained and sandy terrigenous sediments (U4) and Zr-rich terrigenous mud and sand layers intercalated thorough U1, U2 and U3. In general, the arrangement of the sedimentary facies indicates a progressive transition from carbonate fine sediments (U1 & U2) to organic sediments (U3) that gradually change to siliciclastic terrigenous facies (U4) (Figs. S1, S2) with episodic intercalations of Zr-rich mud and/or sands.

After PCA, the elemental composition variation observed thorough the core sediments is summarised in four principal components (PC) which explain 83.4% of the total variance (Table S1). PC1 explains 38.4% of the variance. Rb, Ti, Zn, Fe, V and Ga show high positive loadings (>0.7) and Ca and Sr have a high negative factor loading (<-0.7) (Table S1). Factor scores show that chemical elements with negative loading in PC1 (Ca and Sr) are dominant in units 1, 2 and the first half of unit 3. They show oscillations (minor Ca content) in intervals where Zr-rich siliciclastic (red bands) or organic facies (grey bands) are present (Fig. S2). The upper half of U3 and the entire U4 are characterized by the major presence of positive loading bearing PC1 elements: Rb, Ti, Zn, Fe, V and Ga (Table S1, Fig. S2).

PC2 explains 26.5% of the variance. Cu and Ni show high positive loadings, and Al, Si and K show high negative loadings (Table S1). Factor scores (Table S1 and Fig. S2) are positive in U1 and U3 pointing to a relevant presence of Cu and Ni. In U2 and U4 PC2 shows negative values, more negative in U4 than in U2, indicating a relatively higher Al, Si and K content in the former. PC3 explains 10.2% of the variance. This PC includes only Br with a high negative loading (-0.85) (Table S1). The record of PC3 scores shows negative intervals, with higher Br content, mainly in the lower half of U3 and U4 (Fig. S2).

| Elements | Communalities | PC1 | PC2 | PC3 |
| --- | --- | --- | --- | --- |
| Rb | 0.88 | **0.86** | **0.78** | 0.49 |
| Ti | 0.91 | **0.85** | **0.75** | 0.30 |
| Zn | 0.91 | **0.82** | 0.50 | 0.30 |
| Fe | 0.78 | **0.82** | 0.46 | 0.26 |
| V | 0.64 | **0.78** | 0.40 | 0.25 |
| Ga | 0.79 | **0.72** | 0.30 | 0.24 |
| Pb | 0.74 | **0.67** | 0.25 | 0.15 |
| Cu | 0.72 | 0.30 | 0.24 | 0.11 |
| K | 0.88 | 0.26 | 0.10 | 0.08 |
| Mn | 0.88 | 0.23 | 0.10 | 0.02 |
| Ni | 0.81 | 0.15 | 0.08 | -0.01 |
| Al | 0.94 | 0.05 | -0.01 | -0.01 |
| Si | 0.90 | 0.03 | -0.09 | -0.12 |
| Br | 0.80 | -0.11 | -0.25 | -0.13 |
| Cl | 0.71 | **-0.55** | **-0.76** | -0.37 |
| Sr | 0.93 | **-0.88** | **-0.95** | -0.45 |
| Ca | 0.95 | **-0.92** | **-0.97** | **-0.85** |

Supplementary table 2.1. Results from PCA analysis of the XRF-CS geochemical data from Pla de les Preses core. Communalities and factor load of the analysed 17 elements are presented. Bold values indicate the maximum explained variance (factor loading) for each element.

Supplementary figure 2.2. Main geochemical features of the PdP core indicated by most significant chemical element variation through depth. Most significative lithofacies changes are also indicated (Red bars: volcanic tephra layers; Grey bars: organic intervals; Orange bars: sandy layers). Element abundance is expressed in *counts per second* (cps).


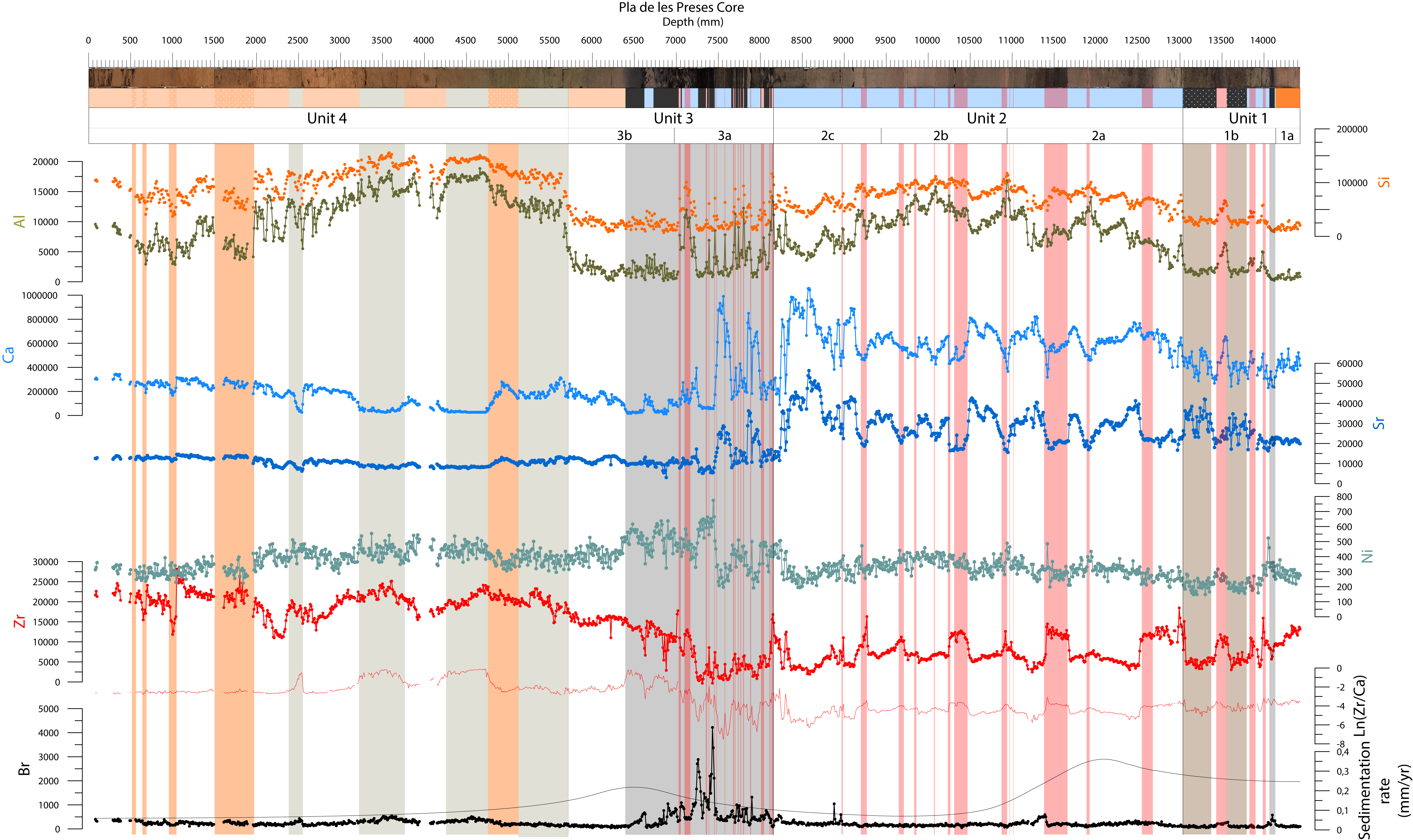


**3. Diatom analysis results.**

The diatom assemblages before and after the tephra deposition in the Pla de Palau indicate a recovery of previous diatom assemblages after tephra deposition. However, the occurrence of the planktonic diatom *Lindavia radiosa (L. radiosa)* only in the post tephra sediment sample would indicate an increase in nutrient availability and a water column. Furthermore, *Diploneis oculata* (*D. oculata*) was the dominant benthic diatom but showed a large relative abundance decrease after the tephra deposition. This would also indicate changes in the distribution and the environmental conditions of benthic mesohabitats.


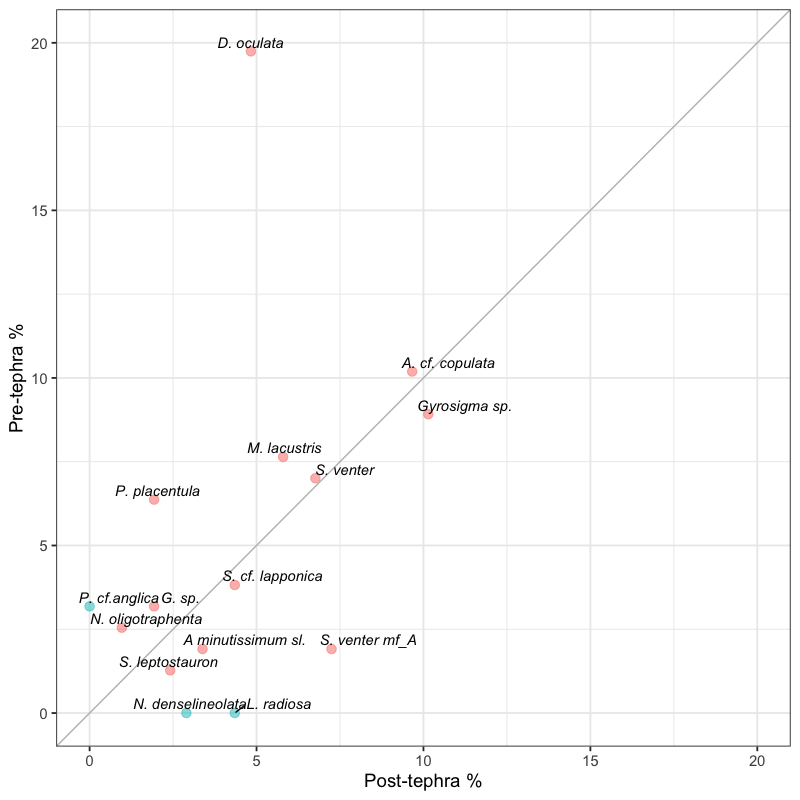


Supplementary figure 2.3**.** 1:1 Plot comparing the most abundant species (> 2%) before and after the Holocene tephra (tephra 6 at 10.35 kyr cal BP) deposition. Points in blue indicate species that did not occur in one of both samples (pre-tephra orpost-tephra sample)**.**

**4. Pla de les Preses paleolake formation and evolution**

The integration of sedimentological and geochemical data enabled the reconstruction of the lake formation processes in Vall d’en Bas valley thorough the Holocene. Seven evolutionary phases could be differentiated.

*4.1. Phase 1: Late Glacial Fluvià river volcanic damming (Unit 1, 14-13.6 ka cal BP; 1445 cm to1300 cm).*

The core sequence starts with brownish mixed carbonate muds (Facies G, B) (see Ca curve in Fig. S2) interpreted as subaerially exposed oxidized shallow lake margin sediments, indicating a possible shallowing trend during Subunit 1a. The shallowing trend supposed the subaerial exposure of previously deposited carbonate mud during Subunit 1a (from >14 until 13.8 ka cal BP). The relatively high content of Cu and Ni (Fig. S2) and Mn in these sediments also point to frequent exposure and vegetation colonization of the sediments in a palustrine environment, since Cu and Ni are usually bonded to hydrous Fe/Mn-oxides and humic substances in lacustrine sediments (e.g. Schmoll and Förstner, 1979; Bendell-Young and Harvey, 1991). The enrichment in Fe and Mn is probably related to vadose oxic conditions on sediments, which involve more favourable conditions for oxidised, less mobile forms, of these elements to form (Chesworth et al., 2006; Jiménez-Espejo et al., 2020).

Afterwards, during Subunit 1b, a deepening upwards facies sequence is observed, shallow vegetated lake margin organic sediments (Facies C) were deposited followed by carbonate-rich lacustrine grey muds (Facies B) denoting shallow water depth. Subunit 1b includes two decimetric intercalations of very coarse bioclastic sand (Facies D) that are interpreted as channel facies that reached the lake margin forming channel-mouth bars in small-scale delta (Wright, 1977). These sandy layers indicate high fluvial and sediment inflow events to the lake pointing to a relatively warm and humid interval at the end of the Bølling-Allerød (BA) as observed in nearby western Mediterranean lacustrine records (Pérez-Obiol and Julià, 1994; Morellón et al., 2009; González-Sampériz et al., 2017).

Although the shallow phase and subaerial exposure inferred from Subunit 1a sediments could be attributed to a decrease of water level/table in the Vall d´en Bas basin due to humidity fluctuations during the BA (GI-1) (e.g. Clark et al., 2012; Morellón et al., 2009), the subsequent deepening trend initiation at *ca.* 13.8 ka cal BP and evolution thorough the Early Holocene includes the Younger Dryas (GS-1) stadial, characterized by the onset of relatively cold and arid conditions in North Iberian Peninsula (e.g. Moreno et al, 2010; Rossi et al., 2018) and specially in nearby Mediterranean lakes (Morellón et al., 2009) that did not show a significative impact in the vegetation in the milder NE Iberia (Pérez-Obiol and Julià, 1994; González-Sampériz et al. 2017). This contradictory fact is attributed to geomorphological changes produced by the intense volcanic activity in GVF between 15.7-13.2 ka cal BP (Puiguriguer et al., 2012; Di Traglia, 2009, 2020). At that time, the Puig Jordà volcano eruption produced large lava flows towards the west, damming the Fluvià river 1 km downstream from the PdP core. The volcanic damming of the Fluvià river provoked the formation of a lake in the Vall d´en Bas valley during latest BA interval and its deepening thorough the Early Holocene, even during the YD, as it is inferred in the PdP core. During the lake formation, in Units 1, 2 and 3, numerous volcaniclastic layers (Facies E) are found intercalated within lacustrine sediments (Fig. S1).

*4.2. Phase 2: Latest Pleistocene PdP shallow lake (Unit 2a, 13.6-12.5 ka cal BP; 1300 cm to 1095 cm).*

During this phase, a progressive deepening of the dammed lake is observed (Unit 2). The sedimentary facies and aquatic proxies from Unit 2a indicate a shallow water lake margin formation. The main facies correspond to carbonate-rich mud (Facies B) with abundant interbedded dark ashy volcaniclastic layers (Facies E) and occasionally centimetre-thick organic sediments (Facies C) with root bioturbation traces (Figs. 4 and 6). The sedimentary facies from Unit 2a indicate the presence of a shallow lake littoral sedimentation (Facies B) with some lower water level periods when vegetation colonization occurred (Facies C). The higher carbonate (Ca) content and the relatively high presence of lithogenic elements indicates major authigenic carbonate production and sedimentation (see Ca curve in Fig. S2) together with a significant fine terrigenous siliciclastic input of fluvial origin. The relative absence of Cu and Ni content points to the absence of significant weathering processes, although the presence of Mn oxides indicate redox condition changes in intermittently flooded soils (e.g. Patrick and Jugsijinda, 1992).

The subsequent deepening trend initiation from Phase 1 continues during Phase 2 and includes the transition from BA (GI-1) to Younger Dryas (GS-1) stadial, that it is characterized by the onset of relatively cold and arid conditions in north Iberian Peninsula (e.g. Moreno et al, 2010; Rossi et al., 2018) and specially in nearby Mediterranean lakes (Morellón et al., 2009). This change to aridity is not observed in PdP core, on the contrary, a deepening trend is observed. As commented before, this contradictory fact is attributed to the water accumulation due to the volcanic damming of the Fluvià river and the onset of a lacustrine water mass in the Vall d´en Bas valley (Fig. S1). Moreover, the sedimentation rates show maximum values during this phase related to the high accommodation space formation due to the progressive deepening of the lake and the high sediment inflow from nearby fluvial input points (e.g. littoral lake deltas). Therefore, volcanic and geomorphological changes affecting the drainage system of the Fluvià river and the subsequent lake formation do not permit to infer regional hydrological and paleoenvironmental variations related to the onset of the YD in the PdP core sedimentary record.

*4.3. Phase 3: Earliest Holocene lake deepening (Unit 2b, 12.5-10.5 ka cal BP; 1095 cm to 945 cm).*

During this phase the stratigraphic Unit 2b was deposited. The sedimentary facies observed are like the ones from Unit 2a, being composed of massive mixed carbonate and siliciclastic mud with intercalated volcaniclastic ash layers (Fig. S1). Siliciclastic clay relative content reaches maximum values during this phase at *ca.*12 ka cal BP, probably denoting less authigenic carbonate formation due to reduced photosynthetic activity of the phytoplankton in colder and/or deeper conditions (Reading, 1996; Morellón et al, 2009). Continuous aggradation of relatively deep fine sedimentary facies indicates that the lake reached its maximum depth during this phase. Since maximum water level in dammed lakes is controlled by the height of the dam, the volcanic lava barrier, it is probable that the lake changed from close to open lake hydrological dynamics during this phase.

Unfortunately, the sedimentary record between 11.7-10.5 ka cal BP, comprising the transition from YD to Early Holocene was lost during coring. Nevertheless, the slightly lower authigenic carbonate production and the ostracod taxa identified suggest relatively cold waters during the first half of the YD as in other Iberian lakes (Morellón et al., 2018). In contrast to other Iberian lake records characterized by low water levels during the YD (Morellón et al., 2018) the Vall d´en Bas paleolake shows deeper conditions due to the continuous infilling of the dammed area.

*4.4. Phase 4: Early Holocene lake shallowing and the 9.3 ka arid event (Unit 2c, 10.5-9.3 ka cal BP; 1095 cm to 815 cm).*

The Early Holocene Unit 2c is composed of the same sedimentary facies present in previous Unit 2 subphases. Carbonate-rich massive mud, with few intercalated volcanic ash layers, shows the highest Ca (carbonate) content thorough the core (Ca from Fig. S2), indicating a maximum authigenic carbonate production during Phase 3. This fact could be related to high organic productivity and photosynthetic activity as it is also observed from a slight but progressive increase in organic matter content related to warmer water temperature as evidenced by the aquatic organisms from this phase.

The observed shallowing process during this Early Holocene phase was culminated by a marked fall of the lake water level and the onset of vegetated littoral peaty sedimentation environment at 9.3 ka cal BP, as evidenced in Unit 3 (Fig. S1). This event is also present in the nearby Lake Banyoles and also represents a rapid change from charophyte-rich shallow carbonate platform facies to littoral peaty wetland conditions (Revelles et al., 2015). Similar drying events were previously attested in western Mediterranean lacustrine records, e.g. in Siles Lake, in the south-eastern Iberian Peninsula (9.3 ka cal BP) (Carrión, 2002), in Fuentillejo Maar (9.2–8.6 ka cal BP) (central Iberian Peninsula; Vegas et al., 2010), in Basa de la Mora (9.3 and 8.8 ka cal BP) (Pyrenees; Pérez-Sanz et al., 2013), in Lake Cerin (9.0 ka cal BP) (Jura Mountains, France; Magny et al., 2011) and Lake Accesa (9.0 ka cal BP) (central Italy; Magny et al., 2007). This lowering in lake water level corresponds with one of the main Holocene rapid climate changes (Mayewski et al., 2004) that is a globally detected dry and cold event centered in 9.3 ka cal BP (Haas et al., 1998; Rasmussen et al., 2006; Fletcher & Zielhofer, 2013), expressed as a decreasing fluvial activity in Mediterranean areas (Magny et al., 2002), dry episodes in the western Mediterranean (Fletcher et al., 2010, 2013; Frigola et al., 2007; Budsky et al., 2019), and episodes of reduced rainfall measured in δ^18^O values of speleothems as in Katerloch Cave (southeastern Alps; Boch et al., 2009), Soreq Cave (Israel; Bar-Matthews et al., 1999) and Cave Victoria (Budsky et al., 2019).

Since the Vall d´en Bas paleolake was already an open lake, the 9.3 ka event most probably supposed a lowering lake level due to a lower water table installation that promoted the fluvial incision of the volcanic dam. A higher incision rate would lead to the lowering of the position of the fluvial outflow point and the subsequent lowering of the water level and the irreversible process of emptying the lake. The concurrence of an arid event and increased incision rate amplified the lake water level reduction around the 9.3 ka event and made it ostensible in the PdP core record.

*4.5. Phase 5: Lake shallowing and the 8.2 ka arid event (Unit 3a, 9.3-8.2 ka cal BP; 8150 cm to 6950 cm).*

During Subunit 3a further shallowing of the lake is observed, shallow vegetated lake margin organic peaty sediments (Facies C) were deposited with fine intercalations of carbonate-rich lacustrine grey muds (Facies B) and abundant millimetre- to centimetre-thick whitish ashy volcaniclastic layers (Facies E) (Fig. S1). The sedimentary facies from Unit 2a indicate the onset of a shallow lake littoral sedimentation (Facies B) with some lower water level periods when vegetation colonization occurred (Facies C). The higher Ca content and the relatively high presence of lithogenic elements indicate major authigenic carbonate production and sedimentation (see Ca in Fig. S2) together with a significant fine terrigenous siliciclastic input of fluvial origin. The intermittent relatively high content of Cu and Ni (Fig. S2) points to the onset of subaerial weathering processes in intermittently flooded soils (e.g. Patrick and Jugsijinda, 1992). PC3, related to the high presence of Br, is related to the organic character of the sediments in Unit 3a (Fig. S1, S2). Bromine is incorporated to organic matter during natural formation of organohalogen compounds that are especially well retained in peaty sediments (Biester et al., 2004 & 2006).

Thus, the shallowing process initiated during Phase 4 (since 9.3 ka cal BP) continued through Phase 5 forming a vegetated lake margin. Moreover, at the end of Phase 4, at *ca.* 8.2 kyr cal BP, the shallowing processes supposed the abrupt disappearance of the lacustrine facies and the nearly continuous subaerial exposure of the core area. Again, the 8.2 ka cal BP event is interpreted as an arid interval that enhanced the lowering of the water table and the lake level and thus promoted the fluvial incision of the volcanic dam. A higher incision rate would led to the lowering of the fluvial outflow point and the subsequent lowering of the water level and the transition from shallow vegetated margin during Unit 3a to the subaerial palustrine environment of Unit 3b. It is noteworthy the presence of abundant thin, millimetre-thick, volcaniclastic ash layers interspersed in organic sediments that indicate the occurrence of numerous volcanic eruptions from 9.3 to 8.3 ka cal BP that constitute the first evidence of Holocene volcanic activity in the GVF and the youngest volcanic activity recorded in the Iberian Peninsula.

As mentioned above, the climatic stability of the Holocene was punctuated by a series of sudden climate changes, particularly during the Early Holocene (e.g. Mayewski et al., 2004). Among these, the ‘8.2 ka event’ has been identified as the largest and most abrupt climatic event of the Holocene (Rohling & Pälike, 2005) and interpreted as the result of an outburst of glacial meltwater from the Laurentide lakes in North America (Lewis et al., 2012). The influx of cold water into the Atlantic Ocean led to a reduction of sea surface salinity and a decline of the Atlantic meridional overturning circulation (AMOC), provoking a reduction in sea surface temperatures (SST) across the North Atlantic (Mary et al., 2017). The cooling effects of this event have been documented in proxies from the Greenland ice cores and across Europe (Rohling & Pälike, 2005; Seppä et al., 2007; Prasad et al., 2009). Short and sharp periods of colder or drier conditions have also been recorded at ~8.2 ka throughout the northern (Alley & Agústsdóttir, 2005; Morrill & Jakobsen, 2005) and southern hemispheres (Dixit et al., 2014; Bustamante et al., 2016).

*4.6. Phase 6: Lake draining (Unit 3b, 8.2-7.1 ka cal BP; 6950 cm to 5700 cm).*

Unit 3b comprises brownish orange sandy siliciclastic mud (Facies F) (Fig. S1) interpreted as oxidized palustrine to fluvial sediments that were subaerially exposed, like Unit 1a. The mud from this unit has a low content of carbonate, and together with the relatively high content of Cu and Ni and Mn in these sediments (Fig. S2), point to incipient weathering due to frequent exposure and vegetation colonization of the sediments in a palustrine environment (e.g. Schmoll and Förstner, 1979; Bendell-Young and Harvey, 1991). The gradual increase of Mn oxides at the top of Unit 3b indicates redox condition changes in intermittently flooded soils (e.g. Patrick and Jugsijinda, 1992) in agreement with the onset of a fluvial sedimentary environment. The sedimentation rate shows a marked maximum during this phase, probably related to the high flooding frequency and fluvial sediment deposition during this phase (Fig. S4). Two dark organic sediment intervals (Facies C), representing the last vegetated lake margin episodes, are present in the basal part of Unit 3a. It is also noteworthy the absence of volcaniclastic sediments in the entire unit.

The shallowing process initiated during Phase 4 (9.3 ka cal BP) culminated in Phase 6 with the draining and disappearance of the lake. At the end of Phase 6, *ca.* 7.1 kyr, a rapid change from lacustrine/palustrine facies to subaerial fluvial sediments (floodplain facies) is observed. This rapid change is broadly coincident with the arid 7.4 kyr event detected in northern Spain (e.g. Pyrenean Basa de la Mora lake, Pérez-Sanz et al., 2013), in Minorca Sea (Frigola et al., 2007) and southern Spain (Jalut et al., 2000; Vegas et al., 2010) and also correlates with a phase of forest decline detected in the Western Mediterranean (Fletcher et al., 2010) in relation to a widespread climatic anomaly (Hou et al., 2019). Thus, the final draining of the PdP lake could be interpreted as promoted by an arid interval that enhanced the lowering of the water table and promoted the fluvial incision and the erosion and disappearance of the volcanic damming. Therefore, the PdP area became again a fluvial environment after 7 metres of lacustrine sediment aggradation due to the Fluvià river valley volcanic damming at *ca.* 13500 yrcal BP.

*4.7. Phase 7: Fluvial aggradation (Unit 4, 7.1 ka cal BP-present; 6950 cm to 0 cm).*

This phase is composed by an alternation of different granulometry siliciclastic sediments, massive greenish mud (Facies H), brownish orange sandy muds (Facies F) and laminated fine sand (Facies G) (Fig. S1). Geochemically they are enriched in typical lithogenic elements from clay minerals and other silicates as quartz and feldspars (Si, Al, K, Ti, Fe, Rb, Zr) (Fig. S2). The geochemical (Al and Zr-rich and Ca-poor) and mineralogical composition of these sediments (Fig. S2) is similar to terrigenous siliciclastic and volcanic sediments analysed in the previous lacustrine sequence, indicating the upstream erosion and resedimentation of older volcaniclastic sediments due to fluvial incision of previous Pleistocene and Holocene fluvio-lacustrine sediments. The relatively high Mn content in most of the unit indicates the presence of a fluctuating near surface water table that also explains the differential formation and preservation of Br and edaphic organic matter in the sediments (Takeda et al., 2018). All this features, together with the absence of aquatic organisms and very poor pollen preservation indicate the fluvial character of the sediments from this phase.

The sedimentation rate decreases gradually thorough the last 6500 years probably due to progressively less intense and/or frequent flooding activity, but this fact is not supported by previous works on Holocene flooding patterns in the eastern Iberian rivers (e.g. Benito et al., 2015a & 2015b). Alternatively, it could be related to ongoing fluvial incision of the Fluvià river in PdP area due to the progressive upstream erosion of the knickpoint formed by the volcanic dam, diminishing the flooding frequency and intensity, and subsequently the sedimentary aggradation in the area.

In summary, the volcanic activity and its geomorphological effect, the Fluvià river valley damming, together with a humid phase during the Early Holocene in the north eastern Iberian Peninsula (Frigola et al., 2007; Benito et al., 2015a & 2015b; Morellón et al., 2018) promoted the formation of a closed lake in PdP area until 9300 yr cal BP. Afterwards, geomorphological processes such as the opening of the lake and the subsequent fluvial incision of the volcanic dam, together with the occurrence of arid events that punctuated the shallowing trend of the lake, promoted the total draining of the lake by 7.1 ka cal BP. Those arid events identified in the PdP core at *ca.* 9.3, 8.2 and 7.1 ka yr BP coincide with the Early Holocene RCCs that promoted the co-occurrence of high-latitude cooling and low-latitude aridity (Mayewski et al., 2004). In the Northern Hemisphere, this RCC intervals correspond to the so-called *Bond events* related to North Atlantic ice-rafting events (Bond et al., 1997 & 2001) that changed the intensity and distribution of atmospheric circulation (e.g. westerlies) over the North Atlantic (Meeker & Mayewski, 2002) that could have controlled rapid humidity and ocean circulation pattern changes in western Mediterranean areas during this RCCs (Frigola et al., 2007; Cisneros et al., 2019).

References

Alley, R.B., Ágústsdóttir, A.M. (2005). The 8k event: cause and consequences of a major Holocene abrupt climate change. *Quaternary Science Reviews*, 24, 1123-1149.

Bar-Matthews, M., Ayalon, A., Kaufman, A. and Wasserburg, G.J. 1999. The Eastern Mediterranean paleoclimate as a reflection of regional events: Soreq Cave, Israel. *Earth Planet. Sci. Lett.*, 166, 85–95.

Bendell-Young, L., Harvey, H.H. 1991. The relative importance of manganese and iron oxides and organic matter in the sorption of trace metals by surficial lake sediments. *Geochim. Cosmochim. Acta*, 56, 1175-1186.

Benito, G., Macklin, M. G., Panin, A., Rossato, S., Fontana, A., Jones, A. F., Machado, M. J., Matlakhova, E., Mozzi, P. and Zielhofer, C. 2015a. Recurring flood distribution patterns related to short-term Holocene climatic variability. *Sci. Rep.*, 5, 16398.

Benito, G., Macklin, M. G., Zielhofer, C., Jones, A. F. and Machado, M. J. 2015b. Holocene flooding and climate change in the Mediterranean. *Catena*, 130, 13–33.

Biester, H., Cortizas, A.M. and Keppler, F. 2006. Occurrence and fate of halogens in mires. *Dev. Earth Surf. Process*. 9: 449–464.

Biester, H., Keppler, F., Putschew, A., Cortizas, A.M. and Petri, M. 2004. Halogen Retention, Organohalogens, and the Role of Organic Matter Decomposition on Halogen Enrichment in Two Chilean Peat Bogs. *Environ. Sci. Technol.*, 38, 1984-1991.

Boch, R., Spötl, C. and Kramers, J. 2009. High-resolution isotope records of Early Holocene rapid climate change from two coeval stalagmites of Katerloch Cave, Austria. *Quat. Sci. Rev.*, 28, 2527-2538.

Bond, G., Showers,W., Cheseby, M., Lotti, R., Almasi, P., de Menocal, P., Priore, P., Cullen, H.,Hajdas, I. and Bonani, G., 1997. A pervasive millennial-scale cycle in North AtlanticHolocene and Glacial Climates. *Science* 278, 1257–1266.

Bond, G., Kromer, B., Beer, J., Muscheler, R., Evans, M.N., Showers, W., Hoffmann, S., Lotti-Bond, R., Hajdas, I. and Bonani, G. 2001. Persistent solar influence on North Atlantic climate during the holocene. *Science*, 294, 2130–2136.

Budsky, A., Scholz, D., Wassenburg, J. A., Mertz-Kraus, R., Spötl, C., Riechelmann, D. F. C., Gibert, L., Jochum, K. P. and Andreae, M. O. 2019. Speleothem δ^13^C record suggests enhanced spring/summer drought in south-eastern Spain between 9.7 and 7.8 ka—A circum-western Mediterranean anomaly? *The Holocene*, 29(7), 1113– 1133.

Bustamante, M. G., Cruz, F. W., Vuille, M., Apaéstegui, J., Strikis, N., Panizo, G., Novello, F., Deininger, A., Sifeddine, A. Cheng, H., Moquet, S., Guyot, J., Santos, R., Segura, H. and Edwards, R. 2016. Holocene changes in monsoon precipitation in the Andes of NE Peru based on δ^18^O speleothem records. *Quat. Sci. Rev.*, 146, 274–287.

Carrión, J.S. 2002. Patterns and processes of Late Quaternary environmental change in a montane region of southwestern Europe. *Quat. Sci. Rev.*, 21(18-19), 2047-2066.

Chesworth W, Martínez Cortizas A and García**–**Rodeja E. 2006. Redox-pH approach to the geochemistry of the Earth´s land surface, with application to peatlands. In: Martini IP, Martínez Cortizas A. and Chesworth W (eds.) *Peatlands: Evolution and Records of Environmental and Climate Changes*. Amsterdam: Elsevier, pp. 175**–**196.

Cisneros, M., Cacho, I., Frigola, J., Sanchez-Vidal, A., Calafat, A., Pedrosa-Pàmies, R., Rumín-Caparrós and Canals, M. 2019. Deep-water formation variability in the north-western Mediterranean Sea during the last 2500 yr: A proxy validation with present-day data. *Glob. Planet. Change*, 177, 56-68.

Clark, P.U., Shakun, J.D., Baker, P.A., Bartlein, P.J., Brewer, S., Brook, E., Carlson, A.E., Cheng, H., Kaufman, D.S., Liu, Z., et al. 2012. Global climate evolution during the last deglaciation. *Proc. Natl. Acad. Sci.*, 109, 1134–1142.

Di Traglia, F. 2020. Hydrogeomorphic and sedimentary response to the Late Pleistocene violent Strombolian eruption of the Croscat volcano (Garrotxa Volcanic Field, Spain). *Med. Geosci. Rev.*, 2, 217-231.

Di Traglia, F., Cimarelli, C., de Rita, D. and Gimeno Torrente, D. 2009. Changing eruptive styles in basaltic explosive volcanism: examples from Croscat complex scoria cone, Garrotxa Volcanic Field (NE Iberian Peninsula). *J. Volcanol. Geotherm. Res.*, 180, 89–109.

Dixit, Y., Hodell, D.A., Sinha, R. and Petrie, C.A. 2014. Abrupt weakening of the Indian summer monsoon at 8.2 kyr B.P. *Earth Planet. Sci. Lett.*, 391, 16-23

Fletcher, W. J. and Zielhofer, C. 2013. Fragility of Western Mediterranean landscapes during Holocene rapid climate changes. *Catena*, *103*, 16-29.

Fletcher, W.J., Sanchez Goñi, M.F., Peyron, O. and Dormoy, I. 2010. Abrupt climate changes of the last deglaciation detected in a Western Mediterranean forest record. *Clim. Past*., 6, 245-264.

Frigola, J., Moreno, A., Cacho, I. and Canals, M. 2007. Holocene climate variability in the western Mediterranean region from a deepwater sediment record. *Paleoceanogr. Paleoclimatol.*, 22(2), PA2209.

González-Sampériz, P., Aranbarri, J., Pérez-Sanz, A., Gil-Romera, G., Moreno, A., Leunda, M., ... and Valero-Garcés, B. 2017. Environmental and climate change in the southern Central Pyrenees since the Last Glacial Maximum: A view from the lake records. *Catena*, *149*, 668-688.

Haas, J.N., Richoz, I., Tinner, W. and Wick, L. 1998. Synchronous Holocene climatic oscillations recorded on the Swiss Plateau and the timberline in the Alps. The *Holocene*, 8(3), 301-309.

Hou, M., Wu, W., Cohen, D. J., Zhou, Y., Zeng, Z., Huang, H., et al. 2019. Evidence for a widespread climatic anomaly at around 7.5–7.0 cal ka BP. *Clim. Past Discuss.*, 1-50.

Jalut, G., Amat, A.E., Bonnet, L., Gauquelin, T. and Fortugne, M. 2000. Holocene climatic changes in the Western Mediterranean, from south-east France to south-east Spain. *Palaeogeogr., Palaeoclimatol., Palaeoecol.*. 160(3), 255-290.

Jiménez-Espejo, F.J., Presti, M., Juhn, G., Mckay, R., Crosta, X., Escutia, C. Lucchi, R.G., Tolotti, R., Yoshimura, T., Ortega Huertas, M., Macri, P., Caburlotto, A. and De Santis, L. 2020. Late Pleistocene oceanographic and depositional variations along the Wilkes Land margin (East Antarctica) reconstructed with geochemical proxies indeep-sea sediments. *Glob. Planet. Change*, 184, 103045.

Lewis, C.F.M., Miller, A.A.L., Levac, E., Piper, D.J.W. and Sonnichsen, G.V. 2012. Lake Agassiz outburst age and routing by Labrador Current and the 8.2 cal ka cold event. *Quat. Int.*, 260, 83-97.

Magny, M., Miramont, C. and Sivan, O. 2002. Assessment of the impact of climate and anthropogenic factors on Holocene Mediterranean vegetation in Europe on the basis of palaeohydrological records. *Palaeogeogr., Palaeoclimatol., Palaeoecol.*, 186, 47-59.

Magny, M., Vannière, B., Calo, C., Millet, L., Leroux, A., Peyron, O., Zanchetta, G., La Mantia, T. and Tinner, W. 2011. Holocene hydrological changes in south-western Mediterranean as recorded by lake-level fluctuations at Lago Preola, a coastal lake in southern Sicily, Italy. *Quat. Sci. Rev*., 30, 2459-2475.

Magny, M., Vannière, B., de Beaulieu, J.-L., Begeot, C., Heiri, O., Millet, L., Peyron, O. and Walter-Simonnet, A.-V. 2007. Early-Holocene climatic oscillations recorded by lake-level fluctuations in west-central Europe and in central Italy. *Quat. Sci. Rev*., 26, 1951-1964.

Mary, Y., Eynaud, F., Colin, C., Rossignol, L., Brocheray, S., Mojtahid, M., Garcia, J., Peral, M., Howa, H., Zaragosi, S. and Cremer, M. 2017. Changes in Holocene meridional circulation and poleward Atlantic flow: the Bay of Biscay as a nodal point. *Clim. Past*, 13, 201–216.

Mayewski, P.A., Rohling, E.E., Stager, J.C., Karlen, W., Maasch, K.A., Meeker, L.D., Meyerson, E.A., Gasse, F., van Kreveld, S., Holmgren, K., Lee-Thorp, J., Rosqvist, G., Rack, F., Staubwasser, M., Schneider, R.R. and Steig, E. J. 2004. Holocene climate variability. *Quat. Res.*, 62, 243-255.

Meeker, L.D. and Mayewski, P.A., 2002. A 1400-year high-resolution record of atmospheric circulation over the North Atlantic and Asia. *The Holocene*, 12, 257–266.

Morellón, M., Aranbarri, J., Moreno, A., González-Sampériz, P. and Valero-Garcés, B.L. 2018. Early Holocene humidity patterns in the Iberian Peninsula reconstructed from lake, pollen and speleothem records. *Quat. Sci. Rev*., 181, 1-18.

Morellón, M., Valero-Garcés, B., Vegas-Vilarrúbia, T., González-Sampériz, P., Romero, Ó., Delgado-Huertas, A., Mata, P., Moreno, A., Rico, M. and Corella, J.P. 2009. Lateglacial and Holocene palaeohydrology in the western Mediterranean region: the lake Estanya record (NE Spain). *Quat. Sci. Rev.* 28, 2582–2599.

Moreno, A., Stoll, H., Jimenez-Sanchez, M., Cacho, I., Valero-Garces, B., Ito, E.and Edwards, R.L.  2010. A speleothem record of glacial (25–11.6 ka BP) rapid climatic changes from northern Iberian Peninsula. *Glob. Planet. Change*, 71, 218-23.

Morrill, C. and Jacobsen, R.M. 2005. How widespread were climate anomalies 8200 years ago? *Geophys. Res. Lett.*, 32, L19701.

Patrick, W. H. and Jugsujinda, A. 1992. Sequential reduction and oxidation of inorganic nitrogen, manganese, and iron in flooded soil. *Soil Sci. Soc. Am. J*., 56(4), 1071.

Pèrez-Obiol, R. and Julià, R. 1994. Climatic change on the Iberian Peninsula recorded in a 30,000-yr pollen record from Lake Banyoles. *Quat. Res.*, 41(1), 91-98.

Pérez-Sanz, A., González-Sampáriz, P., Moreno, A., Valero-Garcés, B., Gil-Romera, G., Rieradevall, M., Tarrats, P., Lasheras-Álvarez, L., Morellón, M., Belmonte, A., Sancho, C., Sevilla-Callejo, M. and Navas, A. 2013. Holocene climate variability, vegetation dynamics and fire regime in the central Pyrenees: the Basa de la Mora sequence (NE Spain). *Quat. Sci. Rev*., 73, 149-169.

Prasad, S., Witt, A., Kienel, U., Dulski, P., Bauer, E. and Yancheva, G. 2009. The 8.2 ka event: Evidence for seasonal differences and the rate of climate change in western Europe. *Glob. Planet. Change*, 67, 218-226.

Puiguriguer, M., Alcalde, G., Bassols, E., Burjachs, F., Expósito, I., Planagumà, L. and Saña, M. 2012. ^14^C dating of the last Croscat volcano eruption (Garrotxa Region, NE Iberian Peninsula). *Geol. Acta*, 10(1), 43–47.

Rasmussen, S. O., Andersen, K. K., Svensson, A. M., Steffensen, J. P., Vinther, B. M., Clausen, H. B., Siggaard-Andersen, M.-L., Johnsen, S. J., Larsen, L. B., Dahl-Jensen, D., Bigler, M., Röthlisberger, R., Fischer, H., Goto-Azuma, K., Hansson, M.E. and Ruth, U. 2006. A new Greenland ice core chronology for the last glacial termination. *J. Geophys. Res.*, 111 (D6), D06102.

Reading, H.G. 1996. Sedimentary Environments Processes, Facies and Stratigraphy. 3^rd^ Edition, Blackwell, Oxford, 689 p.

Revelles, J., Cho, S., Iriarte, E., Burjachs, F., van Geel, B., Palomo, A., Piqué, R., Peña-Chocarro, L. and Terradas, X., 2015. Mid-holocene vegetation history and Neolithic land-use in the Lake Banyoles area (Girona, Spain). *Palaeogeogr., Palaeoclimatol., Palaeoecol.*, 435, 70-85.

Rohling, E.J. and Pälike, H. 2005. Centennial-scale climate cooling with a sudden cold event around 8,200 years ago. *Nature*, 434, 975.

Rossi, C., Bajo, P., Lozano, R.P. and Hellstrom, J. 2018. Younger Dryas to Early Holocene paleoclimate in Cantabria (N Spain): Constraints from speleothem Mg, annual fluorescence banding andstable isotope records. *Quat. Sci. Rev*., 192, 71-85.

Schmoll, G. and Förstner, U. 1979. Chemical associations of heavy metals in lacustrine sediments. *N- Jb. Miner. Abh*., 35(2), 190-208.

Seppä, H., Birks, H.J.B., Giesecke, T., Hammarlund, D., Alenius, T., Antonsson, K., Bjune, A.E., Heikkilä, MacDonald, M.G.M., Ojala, A.E.K., Telford, R. J. and Veski, S. 2007. Spatial structure of the 8200 calyr BP event in northern Europe. *Clim. Past Discuss.*, 3, 165–195.

Takeda, A., Nakao, A., Yamasaki, S. and Tsuchiya, N. 2018. Distribution and speciation of Bromine and Iodine in volcanic ash soil profiles. *Soil Sci. Soc. Am. J.*, 82, 815-825.

Vegas, J., Ruiz-Zapata, B., Ortiz, J.E., Galán, L., Torres, T., García-Cortés,  A., Gil-García, M.J., Pérez-González, A. and Gallardo-Millán, J.L. 2010. Identification of arid phases during the last 50 cal. ka BP from the Fuentillejo maar-lacustrine record (Campo de Calatrava Volcanic Field, Spain). *J. Quat. Sci.*, 25, 1051-1062.

Wright, L.D. 1977. Sediment transport and deposition at river mouths: A synthesis. GSA Bulletin, 88(6), 857-868.
