# Supplementary material for: Socio-ecological impact of monogenetic volcanism in the La Garrotxa Volcanic Field (NE Iberia)
Source: Sci Rep. 2023 May 20;13:8168. doi: 10.1038/s41598-023-35072-0 (PMC10199944; doi:10.1038/s41598-023-35072-0)
Supplement: Supplementary file 3 — Supplementary Information 3. [file 41598_2023_35072_MOESM3_ESM.docx]

***SUPPLEMETARY FILE 3.***

***Summary of the volcanic activity at La Garrotxa Volcanic Field***

Situated in the northeast of the Iberian Peninsula, La Garrotxa Volcanic Field is part of the Catalan Volcanic Zone and one of the provinces of the Neogene-Quaternary alkaline volcanism associated with the European Rift System. It covers about 600 km^2^ and lies between the cities of Olot and
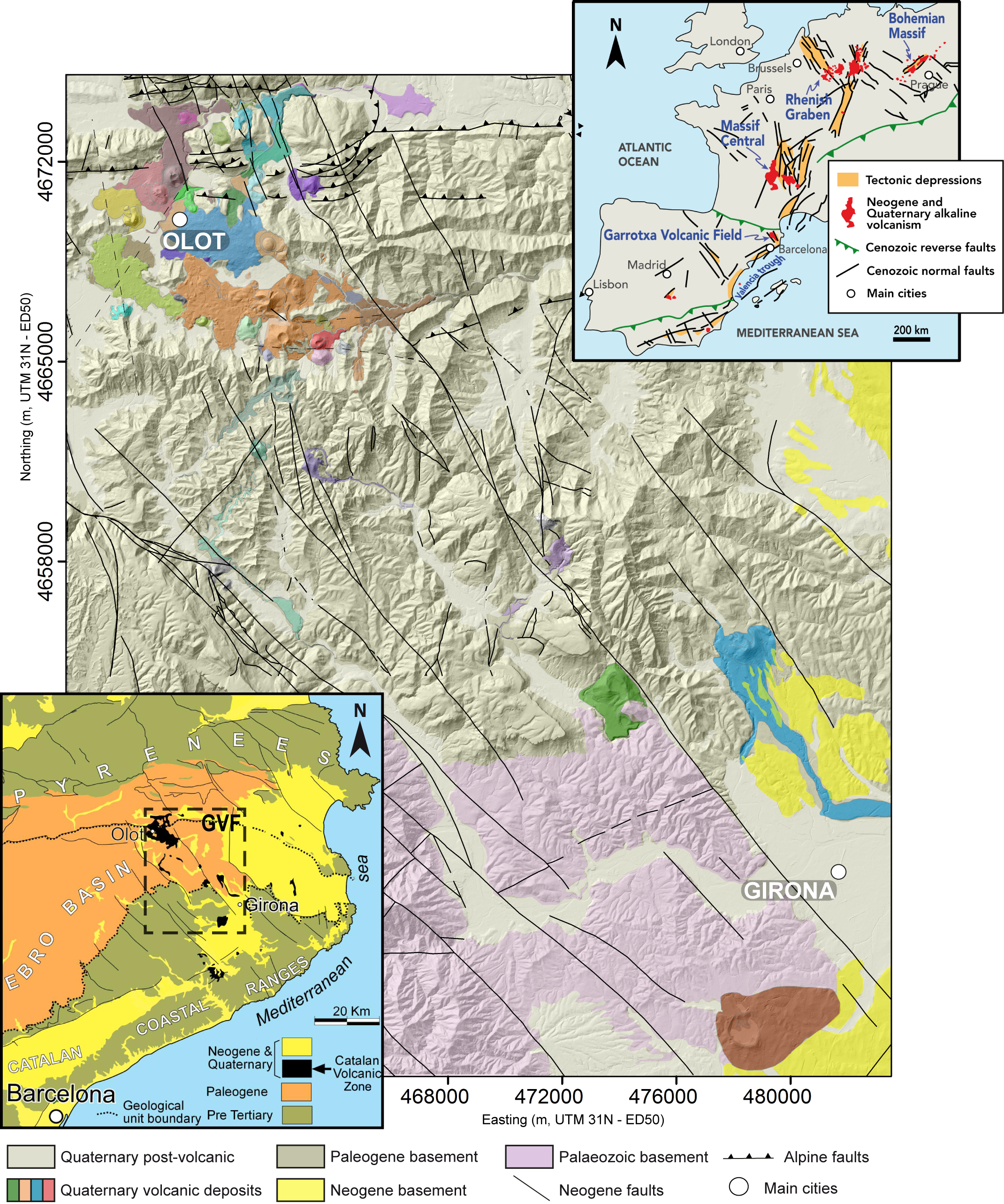
Girona (Martí et al., 2011; Bolós et al., 2014).

***Supplementary figure 3.1.*** Geological setting the study area. Inlet at the upper right corner: Structural map of the European Rift System. Inlet at the lower left corner: Geological map of the NE of Spain. Central image: Geological map of La Garrotxa Volcanic Field (from Martí, J, and Planagumà, Ll., 2016)

This basaltic volcanic field contains over 50 cones (including both cinder and scoria cones), lava flows, tuff rings and maars dating from the Middle Pleistocene to the early Holocene, which rest either on upper Palaeozoic granites and schists or on sedimentary Eocene and Quaternary substrata. Available petrological and geochemical data indicate that this region consists of a suite of intracontinental leucite, basanites, nepheline basanites and alkali olivine basalts, which in most cases represent primary or near-primary magmas, their geochemical characteristics being very similar to analogous petrologic types found in other European Cenozoic volcanic zones.

            La Garrotxa Volcanic Field embraces two geographically distinct zones, the larger area located in the north of the county of La Garrotxa, mostly corresponding to La Garrotxa Volcanic Zone Natural Park, and a southerly area that contains fewer but larger and more complex volcanic edifices (Suppl. Fig. 3.1). Although both correspond to tectonically controlled depressions, the northern zone has substrata consisting of thick layers of Tertiary and Quaternary sediments, whereas the southern zone is underlain by unconsolidated Quaternary sediments in combination with the Palaeozoic basement.

            Volcanic activity in La Garrotxa Volcanic Field is characterised by numerous small cinder cones built during short-lived monogenetic eruptions occurring along tectonic-related volcanic fissures.  The total volume of extruded magma in each eruption was between 0.01 and 0.2 km3 (DRE). Strombolian and phreatomagmatic episodes alternated in most of these eruptions and gave rise to complex stratigraphic sequences with a broad range of pyroclastic deposits. The eruption sequences differ from one cone to another and demonstrate that the eruptions did not follow a common pattern, particularly in cases of magma/water interaction (Martí et al. 2011).

            The existence of this volcanism is linked to the complex geodynamic evolution of the area following the Alpine orogeny that involved great stretching and breakage of the continental lithosphere, thereby allowing the generation of mafic magmas in the mantle and their subsequent ascent and eruption. It is part of the European Cenozoic Rift System that extends from the coast of the North Sea to the Mediterranean and consists of the Spanish Valencia Trough, the Gulf of Lion and Massif Central in France, the Rhine, Ruhr Valley and Leine grabens in Germany, which straddle the river Rhine and cut across the Rhenish Shield, and the Eger Graben in the Bohemian Massif (fig. 1). The age of alkaline basaltic volcanism in the Valencia Through goes from Middle Miocene to Holocene times (Martí et al., 1992; Martí and Bolós, 2019). The most recent and greatest concentration of this volcanism is found La Garrotxa Volcanic Field with volcanic rock ranging from 0.7 Ma to early Holocene.

The evolution of La Garrotxa Volcanic Field is chiefly controlled by two major Neogene faults, the Amer and Llorà faults, oriented NW-SE like most of the major post-Alpine extensional faults that have defined horst and graben structural patterns in NE Iberia. However, most of the eruptive fissures and secondary structural lineaments that control the volcanic activity in La Garrotxa Volcanic Field exhibit a NNW-SSE trend that runs slightly obliquely to the main faults (Bolós et al., 2015).

            The volcanic activity in La Garrotxa Volcanic Field occasioned the accumulation of thick layers of volcanic rocks that, in combination with the particular microclimate of the area, has guaranteed the formation of fertile soils covered by dense vegetation, a process that has helped preserve some of the original volcanic morphologies.

***Volcanic deposits from Pa de les Preses Core***

The sedimentary record from PdP core offers the opportunity to study the volcanic activity in the GVF for the last 14 kyr, and for the first time to extent its activity throughout the Early Holocene, until 8.3 ka cal BP. The sedimentological and geochemical data from the PdP core make it possible to detect numerous tephra layers, mainly composed of millimetre-thick altered ash layers and dark centimetre-thick fine lapilli scoria layers (Supplementary Fig. 3.2).


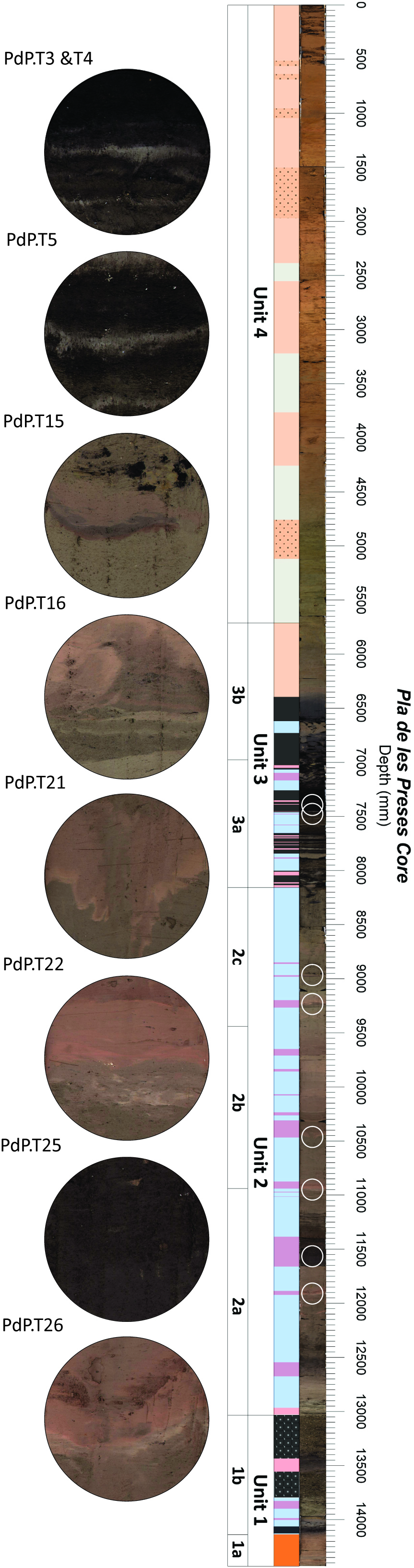


***Supplementary figure 3.2.*** Examples of different type tephra layers present in PdP core.

The vitreous composition and the altered character of most tuffaceous sediments make it difficult to obtain their original mineralogical composition, but the geochemical and sedimentary features clearly point to their allochthonous composition and origin (Fig. 2 in the text). In general, fine ash layers have whitish colours and coarser fine lapilli (or very coarse ash) are black. They have more siliceous composition (clay minerals, quartz and feldspar presence) than autochthonous lacustrine carbonate sediments and are characterized by a red staining reflecting a high Fe content derived from volcanic mineral alteration and iron oxide (hematite) formation (Longman et al., 2023) (Figs. 2 and Suppl. Figures 3.2 & 3.3). Tephra layers are typically characterized by high Zr and low Ca concentrations (Figs. 2 & 3.3), and hence produce higher value intervals in the Zr/Ca ratio curve (Zhang and Liu, 1996) (Fig. 2). Additionally, both elements are known also for carrying textural information (Wu et al., 2020). Zirconium is mainly enriched in heavy mineral species, particularly zircon (ZrSiO4), which is formed by various magmatic (our case) and metamorphic processes (Fralick and Kronberg, 1997). In contrast, Ca is, in this case, indicative of fine-grained authigenic carbonate mud precipitated from carbonate-saturated lacustrine water. Thus, tephra layers in PdP core are geochemically easily inferred using the Zr content through the Ln(Zr/Ca) ratio (Fig. 2 & 3.3) in high-resolution XRF-CS records (Toro et al., 2013; Wu et al., 2020). Thirty tephra layers grouped in 3 different temporal clusters could be differentiated between 13.6 and 8.3 ka cal BP (Fig. 4 and Table III).

Concerning the origin of the tephra and lapilli layers, future geochemical evidence will lead to elucidate its autochthonous (GVF) or allochthonous origin. Different western European volcanic regions in Iceland and Greenland (e.g. Housley et al., 2012), France (Chaîne des Puys; Nowell, 2008; Miallier et al., 2010), Italy (e.g. Milia and Torrente, 2020) and Canary Islands (Rodriguez-Gonzalez et al., 2018) experienced volcanic eruptions during the Late Plesitocene and/or the Holocene. Nevertheless, none of them covers the complete temporal interval or coincides in chronology with the ages of the tephra levels detected in the PdP Core. This fact together with the presence of relatively proximal lapilli layers and tremor-related deformation structures, and the previous volcanological studies demonstrating the presence of phreatomagmatism in the last GVF eruptions (Cimarelli et al., 2010 & 2013; Martí et al., 2011) support the autochthonous character of the tephra and lapilli layers observed in the PdP core (Fig. 3.3).


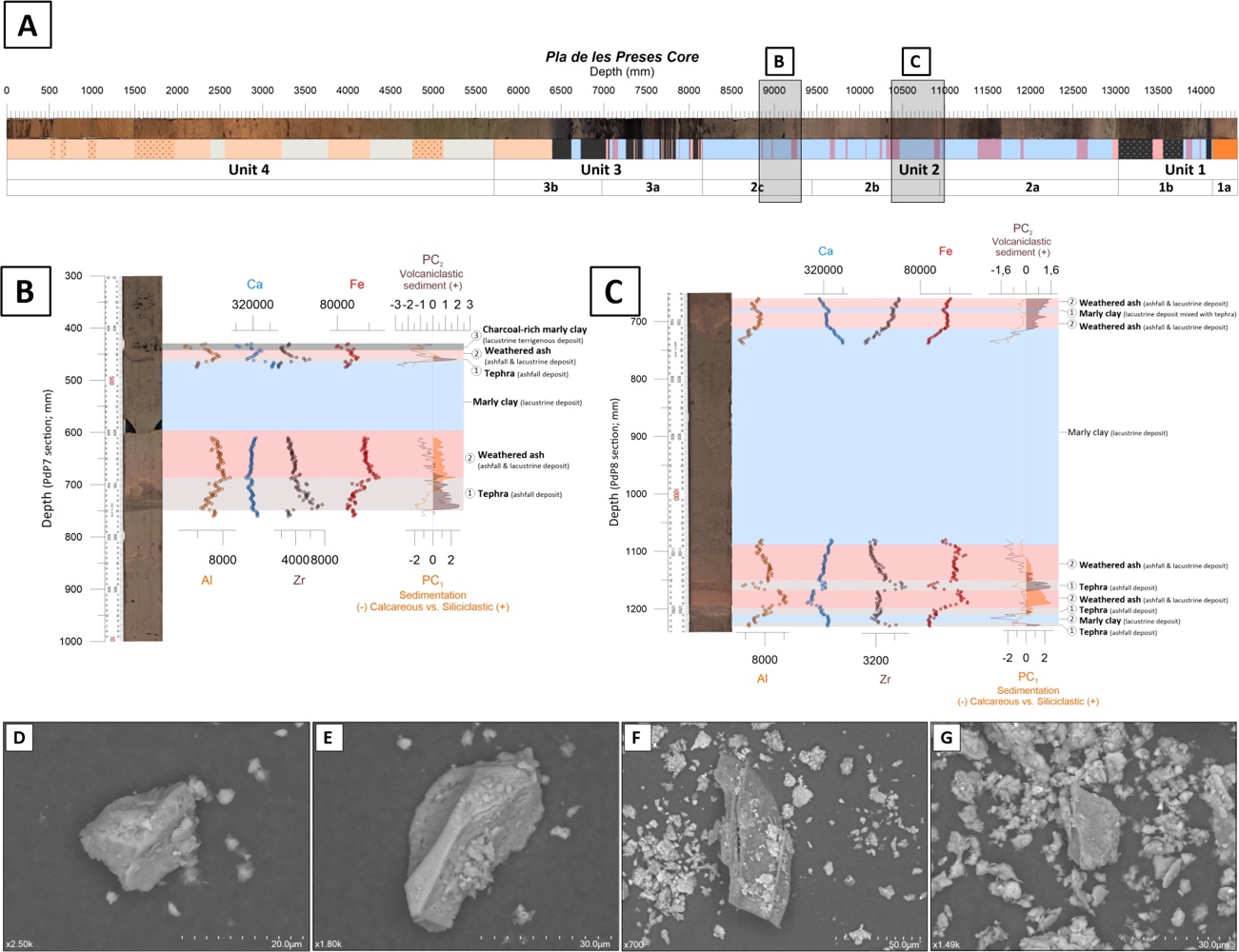


***Supplementary figure 3.3.*** High-resolution geochemical XRF-CS analysis of Early Holocene tephra layers in PdP core. A-C) PCA analysis discriminates lacustrine sediment (carbonate and siliceous) PC_1_ from allochthonous volcaniclastic sediment (tephras) PC2. Zr-enriched sediment correspond to relatively coarse volcanic tephras, and Fe-enriched sediments are lacustrine fine sediments containing high-content of altered volcanic ash. D-G)SEM photographs of basaltic tephra fragments. All angular pyroclasts correspond to interior fragments from broken vesicular ash fragments with large, well-developed vesicles and very thick walls between vesicles. Grain surfaces are covered of alteration products and secondary minerals.

***References***

Bolós X, Planagumà L, Martí J (2014) Volcanic stratigraphy and evolution of the Quaternary monogenetic volcanism in the Catalan Volcanic Zone (NE Spain). Journal of Quaternary Science 29(6): 547–560.

Bolós X, Martí J, Becerril L, Planagumà LL, Grosse P, Barde-Cabusson S (2015) Volcano-structural analysis of La Garrotxa Volcanic Field (NE Iberia): implications on the plumbing system. Tectonophysics 642: 58–70. http://dx.doi.org/10.1016/j.tecto.2014.12.013

Cimarelli, C., Di Traglia, F., de Rita, D., Torrente, D.G. (2013). Space–time evolution of monogenetic volcanism in the mafic Garrotxa Volcanic Field (NE Iberian Peninsula). Bull Volcanol., 75(11), 1–18.

Fralick, P., Kronberg, B. I. (1997). Geochemical discrimination of clastic sedimentary rock sources. Sedimentary Geology, 113, 111–124.

Housley, R.A., Lane,, C.S, Cullen, V.L., Weber, M.-J., Riede, F., Gamble, C.S., Brock, F. (2012). Icelandic volcanic ash from the Late-glacial open-air archaeological site of Ahrenshöft LA 58 D, North Germany. Journal of Archaeological Science, 39, 708-716.

Longman, J., Dunlea, A.G., Böning, P., Palmer, M.R., Gernon, T.M., McManus, J., Manners, H.R., Homoky, W.B., Pahnke, K. (2023). Release of tephra-hosted iron during early diagenesis fingerprinted by iron isotopes. Earth and Planetary Science Letters, 605, 118016.

Martí, J, Planagumà, Ll. (Edrs) (2016) La Garrotxa Volcanic Field: Example of sustainable volcanic landscape managementSpringer Geoheritage, Geoparks and Geotourism Conservation and Management Serie, 136 pp. ISBN: 978-3-319-42078-3 (Print) 978-3-319-42080-6 (Online).

Martí, J., Bolós, X., (2019) The Neogene-Quaternary alkaline volcanism of Iberia, in Quesada, C. and Oliveira, J. T. (eds) " The Geology of Iberia: A Geodynamic Approach", Volume 4: Cenozoic Basins, Springer. Regional Geology Reviews, https://doi.org/10.1007/978-3-030-11190-8_6

Martí, J., Planaguma, L., Geyer, A., Canal, E., Pedrazzi, D. (2011). Complex interaction between Strombolian and phreatomagmatic eruptions inthe Quaternary monogenetic volcanism of the Catalan VolcanicZone (NE of Spain). J. Volcanol. Geotherm. Res., 20(1–4), 178–193.

Martí J, Mitjavila J, Roca E, Aparicio A (1992) Cenozoic magmatism of the Valencia Trough (Western Mediterranean): relation between structural evolution and Volcanism. Tectonophysics 203: 145–166.

Miallier, D., Boivin, P., Deniel, C., Gourgaud, A., Lanos, P., Sforna, M., Pilleyre, T. (2010). The ultimate summit eruption of Puy de Dôme volcano (Chaîne des Puys, French Massif Central) about 10,700 years ago. Comptes Rendus Geoscience, 342, 847-854.

Milia, A., Torrente, M.M. (2020). Space-time evolution of a volcanic field in an extended region: the example of the Campania margin (Italy). In: B. De Vivo, H.E. Belkin, G. Rolandi (Eds.), Vesuvius, Campi Flegrei, and Campanian Volcanism. Elsevier, pp. 297-321.

 Nowell, D. (2008). [The Chaîne des Puys volcanoes of the Auvergne, France](https://www.researchgate.net/publication/230357466_The_Chaine_des_Puys_volcanoes_of_the_Auvergne_France), Geology Today, 24, 6, 231-238.

Rodriguez-Gonzalez, A., Perez-Torrado, F.J., Fernandez-turiel, J.L., Aulinas, M., Paris, R., Moreno-Medina, C. (2018). The Holocene volcanismo f Gran Canaria (Canary Islands, Spain). Journal of Maps, 14(2), 620-629.

Toro, M., Granados, I., Pla, S., Giralt, S., Antoniades, D., Galán, L., Martínez Cortízas, A., Lim, H.S., Appleby, P.G. (2013). Chronostratigraphy of the sedimentary record of Limnopolar Lake, Byers Peninsula, Livingston Island, Antarctica. Antarctic Science, 25, 198-212.

Wu, L., Wilson, D. J., Wang, R., Yin, X., Chen, Z., Xiao, W., Huang, M. (2020). Evaluating Zr/Rb ratio from XRFscanning as an indicator of Grain-Size variations of glaciomarine sediments in the Southern Ocean. Geochemistry, Geophysics, Geosystems, 21, e2020GC009350.

Zhang, W., Liu, J. (1996). The composition of volcanic ash from ice core of Collins Cap, King George Island of Antarctica. Acta Petrologica Sinica, 12, 434–445.
